# Expression of non-protein-coding antisense RNAs in genomic regions related to autism spectrum disorders

**DOI:** 10.1186/2040-2392-4-32

**Published:** 2013-09-04

**Authors:** Dmitry Velmeshev, Marco Magistri, Mohammad Ali Faghihi

**Affiliations:** 1 Department of Psychiatry and Behavioral Sciences and Center for Therapeutic Innovation, University of Miami Miller School of Medicine, 1501 NW 10th Ave., BRB-407, Miami, FL 33136, USA

**Keywords:** Autism, ASDs, Epigenetics, lncRNAs, NATs, ncRNAs

## Abstract

**Background:**

Autism spectrum disorders (ASD) manifest with neurodevelopmental phenotypes including communicative, social and behavioral impairments that affect as many as 1 in 88 children. The majority of autism cases have no known genetic cause, suggesting complex genetics of the disorder, but a few genes of large effect have been identified.

**Methods:**

In order to identify novel ASD genetic correlates, we investigated non-protein coding RNAs (ncRNAs) which are abundantly transcribed from the human genome, enriched in the brain, and have been implicated in neurodevelopmental disorders. Using an algorithm that we developed, we examined a publicly available transcriptomics database, AceView, to identify the natural antisense transcripts (NATs) that overlap with known autism-related genes. We validated the presence and differential expression of NATs in different brain regions of ASD and control brains using qRT-PCR. Additionally, we investigated the subcellular localization of these transcripts in a neuronal cell line using RNA-sequencing (RNA-seq).

**Results:**

We found noncoding antisense RNA transcripts at approximately 40% of loci previously implicated in ASD. We confirmed the expression of 10 antisense RNAs in different postmortem human brain tissues. The expression of five antisense transcripts was found to be region-specific, suggesting a role for these ncRNAs in the development and function of specific brain regions. Some antisense RNAs overlapping suspected ASD genes exhibited concordant expression relative to their sense protein-coding genes, while other sense-antisense pairs demonstrate a discordant relationship. Interestingly, the antisense RNA corresponding to the *SYNGAP1* locus (*SYNGAP1*-AS) was found to be differentially expressed in brain regions of patients with ASD compared to control individuals. RNA-seq analysis of subcellular compartments from SH-SY5Y human neuroblastoma cells demonstrated that antisense RNAs to ASD candidate genes are predominantly expressed in the nucleoplasmic or chromatin compartments, implying their involvement in nuclear-associated processes.

**Conclusions:**

Our data suggests that NATs are abundantly expressed from ASD-related loci and provide evidence for their roles in target gene regulation, neurodevelopment and autism pathogenesis. This class of RNA should therefore be considered in functional studies aimed at understanding genetic risk factors for ASD.

## Background

Autism spectrum disorders (ASD) are heterogeneous neurodevelopmental disorders, both in terms of clinical manifestations and genetic risk factors [1]. Disease frequency among siblings of affected children is approximately 2% to 8%, which is much higher than the prevalence rate of the general population and monozygotic twins have 60% concordance for classic autism and 92% for broader autistic phenotypes, indicating strong genetic inheritance as the predominant causative agent [2]. Genetic studies show that ASD can arise from rare, but highly penetrant, mutations and genomic imbalances [3,4] with more than a hundred disease-associated genes and genomic loci having been reported [5,6]. Such mutations may contribute to ASD etiology by affecting conventional genes directly or indirectly by altering the function of non-protein coding RNAs (ncRNAs) expressed in the same genomic loci. Recent evidence has implicated such ncRNAs in neurodevelopmental and neurodegenerative disorders including autism [7-15].

Large transcriptomic consortiums such as ENCODE [16] and FANTOM [17,18] have demonstrated that the human genome is pervasively transcribed and that the primary output are ncRNAs. Through diverse mechanisms, these ncRNAs control protein production and function at multiple levels, including epigenetic control of their corresponding or distant loci [19,20], alteration of localization, stability or processing of targets [20,21], or by modulating translational efficiency by binding to the 3’ UTR of transcripts, as in the case of microRNAs [22,23]. Natural antisense transcripts (NATs) are a conserved class of long (>200 nt in length) ncRNA molecules that are transcribed from the opposite DNA strand of a sense RNA partner with which they have sequence complementarity [18,24]. Such antisense RNAs can exert *cis*-regulatory functions to increase (concordant) or decrease (discordant) expression levels of their corresponding sense mRNA [21]. The gene regulator functions can also work *in trans* by affecting genes from distant genomic loci.

Here, we developed an algorithm to mine existing public transcriptomic repositories for the presence of NATs that are produced from ASD candidate genes. We believe that ncRNA information processing systems involving such transcripts represent a critical but under-appreciated dimension of the cell machinery that must be considered in order to identify pathological events and facilitate novel therapeutic development strategies for ASD.

## Methods

### Ethics statement

The University of Miami Institutional Review Board has deemed this study exempt from the full review due to the use of de-identified human post-mortem brain samples, with no possibility to track back the identity of the donors. There is no animal study involved in this paper.

### Postmortem brain tissue and RNA extraction

Tissue samples were provided by the National Institute of Child Health and Development (NICHD) at the University of Maryland. A complete description of the samples is provided in Additional file 1: Table S2.

For RNA extraction ~100 mg of brain tissue was lysed in trizol (Life Technologies), 200 μL of chloroform were added and the sample was incubated at room temperature for 10 minutes. The samples were then centrifuged for 20 minutes at 4°C. The supernatant (aqueous phase) was then transferred to a new tube containing 1.5 volumes of 100% ethanol. The ethanol/RNA mixture was then loaded onto a RNeasy column (Qiagen) and purified as per the manufacturer’s instructions, including on-column DNase treatment. Typical yields from both non-ASD and Autism subjects were about 10–12 μg of total RNA from 100 mg of tissue.

### Primer design

Primers were designed using Primer 3 software with the sequences from AceView and synthesized by Integrated DNA Technologies (Additional file 2: Table S3). Primers were designed for a splice junction when possible; when primers were designed for an exon they were designed either for a region of the antisense transcript that does not overlap the sense gene or for a region where the antisense overlaps an intron of the sense transcript (Additional file 3: Figure S1). In these cases, strand-specific quantitative real-time RT-PCR was utilized to avoid amplifying the transcript encoded on the opposite strand of DNA.

### Quantitative real time RT-PCR (qRT-PCR)

For qRT-PCR, total RNA was reverse transcribed using the High-Capacity cDNA Reverse Transcription Kit (Life Technologies). The cDNA was then diluted 1:5 and was used as a template for both SYBR Green (Life Technologies, 4368706) and TaqMan qPCR using the ABI 7900 (Life Technologies). TaqMan probes for human *PGK1* from Life Technologies (Hs00943178_g1) were used to measure gene expression of the endogenous control. Three technical replicates were performed for each reaction. No-template controls were included in each reaction and the melting curve was analyzed to assess the specificity of each primer (Additional file 4: Appendix 1). In case the primers were designed for a single exon and did not span a splice junction, appropriate no-RT controls were used to avoid including samples contaminated with DNA. The results of the quantitative real-time RT-PCR were analyzed with SDS 2.3 software from Life Technologies.

### Strand-specific qRT-PCR

To perform strand-specific measurement of antisense transcript expression, we designed primers for a region of antisense transcript that overlaps with an intron or the promoter of the sense gene. Next, we used one-step RNA-to-Ct SYBR Green Kit (Life Technologies, 4389986). We performed reverse transcription (RT) step in a 384-well optical plate using reverse primers to specifically reverse-transcribe antisense RNA and to exclude the possibility of measuring the expression of the sense pre-mRNA. Samples were then incubated at 95°C for 5 minutes to inactivate the reverse transcriptase enzyme. Forward primers were then added to the reaction and quantitative PCR was performed on the same plate. We included no-RT control and no-template controls for each set of primers to control for non-specific binding.

### Statistical analysis

For all qRT-PCR reactions, three technical replicates were performed. To compare the expression of antisense RNAs across the three brain regions, GraphPad prism software was used to perform ANOVA followed by Tukey post-hoc test. *A p* value of below 0.05 was considered as statistically significant. The Student’s *t*-test was used to compare the expression between the normal brain and ASD.

### Cellular fractionation

SH-SY5Y cells were fractionated using a modified NE-PER Kit (PIERCE) to isolate RNA from the cytosol, nucleoplasm and chromatin. Briefly, the cells were collected and washed twice with PBS. Cell membranes were lysed using a hypotonic buffer and cells were ultra-centrifuged to pellet nuclei, and the cytosol was recovered from the supernatant. Nuclei were further lysed and centrifuged in order to pellet the insoluble chromatin and recover the nuclear extract in the supernatant. The insoluble chromatin pellet was solubilized in PBS with mild sonication. RNA was extracted from each of the three compartments using a combination of two protocols: Trizol LS (Invitrogen) and RNeasy Mini Kit (QIAGEN). Each sample was dissolved in the appropriate amount of Trizol LS (1 mL for 300 μL sample) and incubated for 10 min at room temperature. Chloroform (200 μL for 1 mL Trizol) was added to the mix and the sample was centrifuged for 20 minutes at 4°C. The aqueous phase of the supernatant was transferred into a new tube and mixed with 1.5 volumes of absolute ethanol. The sample was then loaded onto the cartridge provided by the QIAGEN kit and on-column DNase treatment was performed as per the manufacturer’s protocol. RNA quality was verified using the Agilent Bioanalyzer RNA6000 nano kit.

### Library preparation

RNA samples were prepared for directional RNA sequencing using a modified version of the Illumina sample preparation protocol. Briefly, 1 μg of total RNA was processed using Ribo-ZeroTM rRNA Removal Kits to remove ribosomal RNAs. Ribosome-depleted RNA was treated with phosphatase before being treated with T4 polynucleotide kinase (PNK). PNK-treated RNA was then purified with the QIAGEN RNeasy column purification kit and 3’ and 5’ RNA adapters were ligated to both ends of the RNA in separate reactions. Next, the RNA was reverse transcribed and PCR amplified. PCR products were purified using AMPure beads. RNA sequencing libraries were validated using the Agilent Bioanalyzer High Sensitivity DNA kit and sequenced using the Illumina HiSeq2000 platform at the Genomics sequencing core at the University of Miami. Each sample was run in a single flowcell to increase depth of sequencing.

### RNA-seq analysis

The sequencing reads were pre-processed with a custom Python script to trim library adapters. This allowed the generation of 62,500,000 reads per sample on average, which provided an acceptable coverage and sequencing depth. The trimmed reads were then aligned to the human transcriptome assembly GRCh37 from ENSEMBL using TopHat version 2.0.4 [25]. TopHat was run with default parameters and Samtools [26] were used to calculate the alignment statistics for each sample. The bam files generated with TopHat were further used as input for Cufflinks [27] to perform *ab initio* transcriptome assembly. The assembled fragments were then annotated using the Cuffcompare module of Cufflinks and AceView database file as a reference. The fragments that originated from introns and incompletely spliced RNAs were filtered out, and Fragments Per Kilobase of transcript per Million reads Mapped (FPKM) values for fragments transcribed from each locus were added to obtain locus expression.

## Results

### Bioinformatic identification of ASD-related noncoding RNAs

AceView is a transcriptome database created and supported by the National Center for Biotechnology Information (NCBI) that represents a curated non-redundant collection of RNA transcripts derived from public cDNA collections (mRNAs from GenBank or RefSeq, and single pass cDNA sequences from dbEST and Trace) [28]. AceView also includes information on tissue-specific expression for transcripts and is an excellent source of transcriptomic data for high-throughput genome-wide studies.

**Figure 1:**
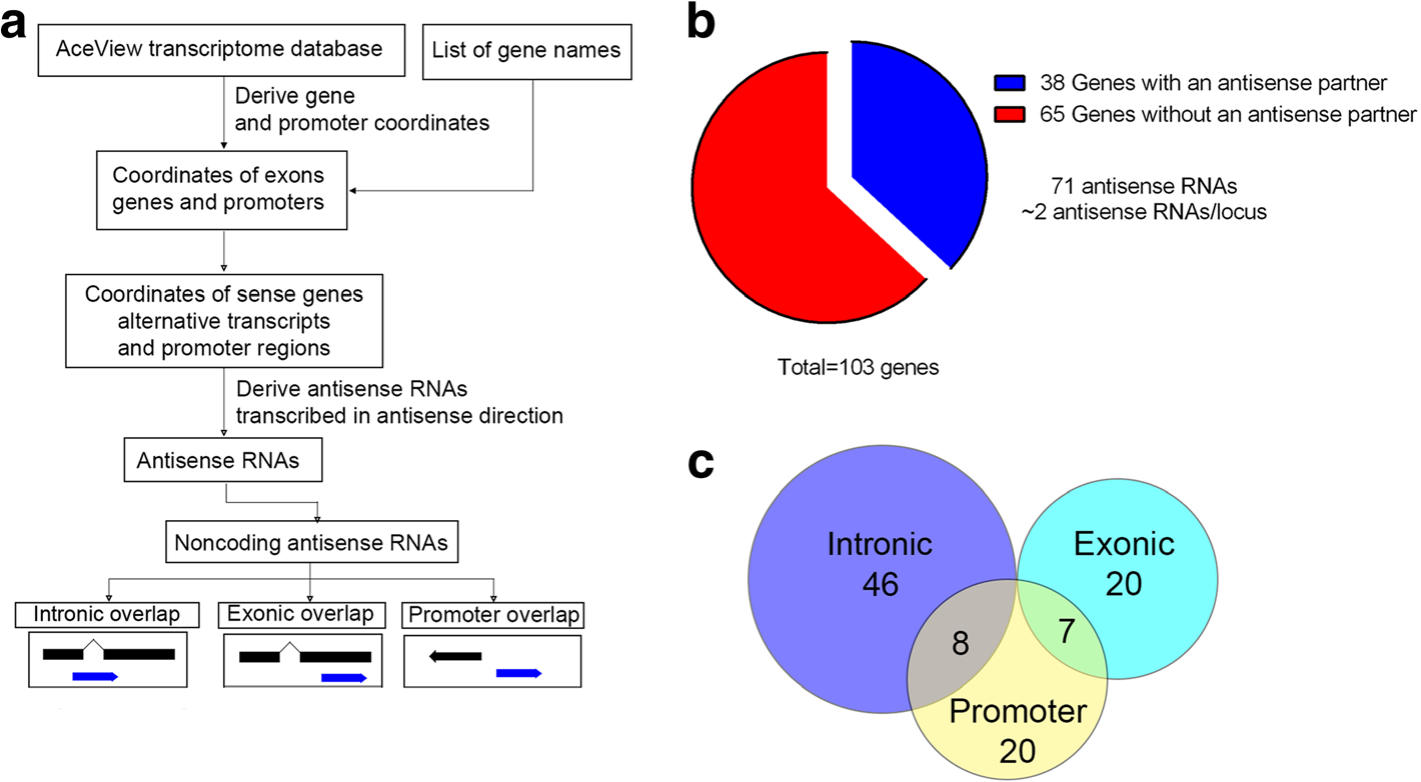
**Bioinformatics identification of NATs expressed from ASD-related loci. (a)** Bioinformatics pipeline used for the identification of noncoding antisense RNAs. **(b)** Antisense noncoding RNAs to 103 ASD-related genes derived with our bioinformatics pipeline. Seventy-one noncoding antisense RNAs were identified overlapping 38 of 103 analyzed genes; thus, each sense gene has ~two antisense RNAs on average. **(c)** Distribution of noncoding antisense RNAs to ASD-related genes based on the type of sense-antisense overlap. The majority of the antisense RNAs overlaps an intron of the sense gene but do not have an overlap with the mature sense transcript (intronic overlap). Approximately equal numbers of antisense RNAs overlap an exon of the sense transcripts (exonic overlap) or gene promoters (promoter overlap); some of the antisense transcripts with intronic or exonic overlap have mixed classifications and can also overlap the promoter regions of their sense partners.

We have developed a bioinformatics pipeline to mine the AceView and to perform high-throughput searches of noncoding RNAs in the antisense orientation to genes of interest (Figure 1a). This pipeline uses information on the genomic coordinates of transcripts, their exonic structure and their coding potential that is contained in the gene transfer format (GTF) files downloaded from the AceView website, to perform a simultaneous search for non-protein-coding antisense RNAs. The program first retrieves, from the AceView database, the exonic coordinates of all alternative transcripts corresponding to a user-provided list of genes. The information of exonic structure of sense transcripts is utilized to obtain AceView transcripts that are in antisense conformation and determine the type of sense-antisense overlap. Next, coding antisense transcripts are filtered out preserving only non-protein-coding transcripts. We utilized this pipeline to investigate the presence of antisense transcripts overlapping 103 genes, mutations of which were causally implicated in ASD [5]. This gene list was selected for our bioinformatics analysis as it was manually annotated by examining existing medical literature and published in a peer-reviewed journal, provided both the scope and confidence in the quality of the analysis [5]. Thus, using this fairly accurate list of genes we were able to identify at least one noncoding antisense RNA partner for 38 of the examined genes (37%) (Additional file 5: Table S1). Overall, 71 noncoding RNA loci were identified, yielding two antisense partners per sense gene on average (Figure 1b). These antisense RNAs represent three structural classes based on the position of the antisense transcript with respect to the sense gene: intronic overlap, exonic overlap and promoter overlap (Figure 1a and c). Therefore, a significant number of gene loci with ASD candidate genes have one or more noncoding antisense transcripts that may contribute to ASD pathophysiology and are henceforth referred to as ASD-NATs.

### ASD-NATs are expressed in human brain tissues

Of the 71 identified ASD-NATs, 18 were selected for qRT-PCR validation studies using commercially available RNAs from total brain extract, frontal cortex, and cerebellum. The expression of 12 transcripts was confirmed in at least one brain region or in the total brain extract (Table 1). Notably, the antisense transcript of *FOXG1* (*FOXG1*-AS) was found in the cortex but not in the cerebellum, implying a certain level of region-specificity in the expression of some ASD-NATs. *FOXG1* encodes a transcription factor thought to play a role in the development of the cortex [29,30] and mutations in this gene have been reported in a variety of neurodevelopment disorders and higher-order brain function [31].

**Table 1:** Antisense RNAs to ASD-related genes expressed in the human brain

**Sense gene name**	**Antisense gene coordinates**	**Antisense AceView name**	**Antisense type**
FOXP1	3:71630795–71678203,1	chyrarbu	exonic; promoter
ZNF81	X:47765952–47764918,-1	zoyfoy	intronic
SYNGAP1	6:33422342–33405140,-1	kleefloybu	exonic; promoter
CACNA1C	12:2781443–2777666,-1	kirare	exonic
NIPBL	5:36876787–36864527,-1	LOC646719	promoter
VPS13B	8:100026175–100008986,-1	speeshor	promoter
NHS	X:17755214–17658171,-1	kiro	exonic
DHCR7	11:71159652–71163207,1	steymor	intronic; promoter
LAMP2	X:119572593–119576511,1	werkoy	exonic
PTEN	10:89631419–89630176,-1	kloloy	intronic
FOXG1	14:29234525–29194448,-1	sachawbu	promoter
PQBP1	X:48758712–48758117,-1	foyker	exonic; promoter

**Figure 2:**
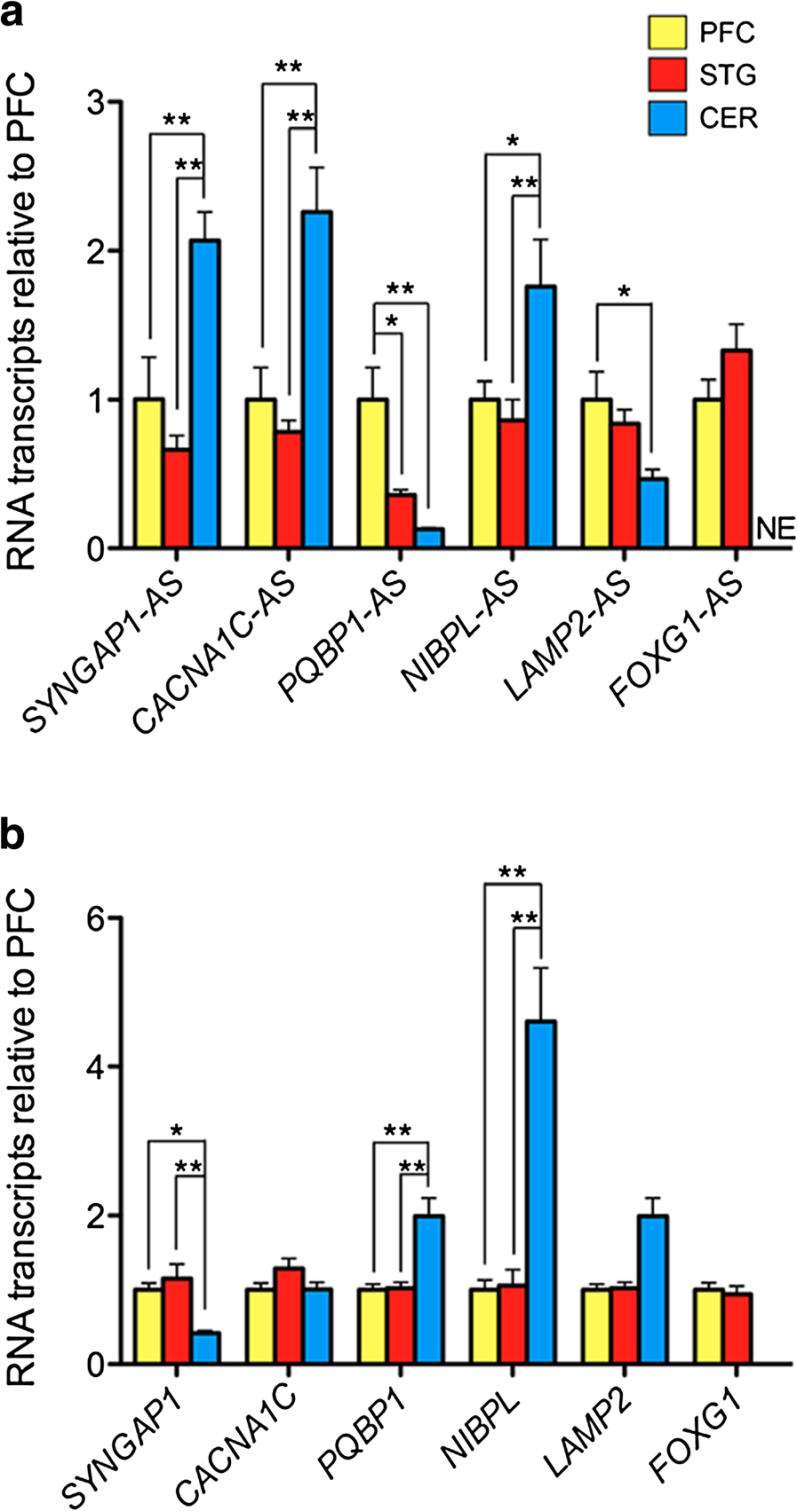
**ASD-related NATs and mRNA expression in different human brain regions.** qRT-PCR analysis of ASD**-**related NATs **(a)** and corresponding mRNA **(b)** in the prefrontal cortex (PFC), superior temporal gyrus (STG) and cerebellum of non-ASD human postmortem brain. Transcript expression is normalized to *PGK1*. Strand-specific qRT-PCR was used to measure expression of *SYNGAP1*-AS and *PQBP1*-AS. *-*P* <0.05, **-*P* <0.01, Tukey’s post-hoc test; NS: not significant, NE: not expressed.

To expand these studies, we compared expression levels of ASD-NATs across multiple brain regions using a cohort of human postmortem brain samples provided by the NICHD at the University of Maryland. RNA was extracted from three brain regions; the prefrontal cortex (PFC), superior temporal gyrus (STG), and cerebellum (Additional file 1: Table S2). We observed that 9 out of the 10 ASD-NATs were detectable in all brain regions, except *FOXG1*-AS, which was detected in all PFC and STG samples but none of the cerebellar samples (Figure 2a and Additional file 6: Figure S3). This finding corroborates our initial analysis using commercial RNA from the frontal cortex, and confirms region-selective expression of *FOXG1*-AS.

Our data demonstrate that the majority of noncoding antisense RNAs from ASD-related loci are expressed in the human brain and suggest the possibility that certain ASD-NATs may have region-dependent patterns of expression reflecting their biological functions.

### ASD-NATs are differentially expressed in human brain regions

Many ncRNAs are dynamically regulated during differentiation and exhibit tissue- and cell type-specific patterns of expression with proposed functions and mechanisms far more complex than originally anticipated [32-36]. Temporal and spatial expression of many long ncRNAs appears to be crucial for proper CNS development and neurological functioning through the precise regulation of a variety of biological processes [37-39].

**Figure 3:**
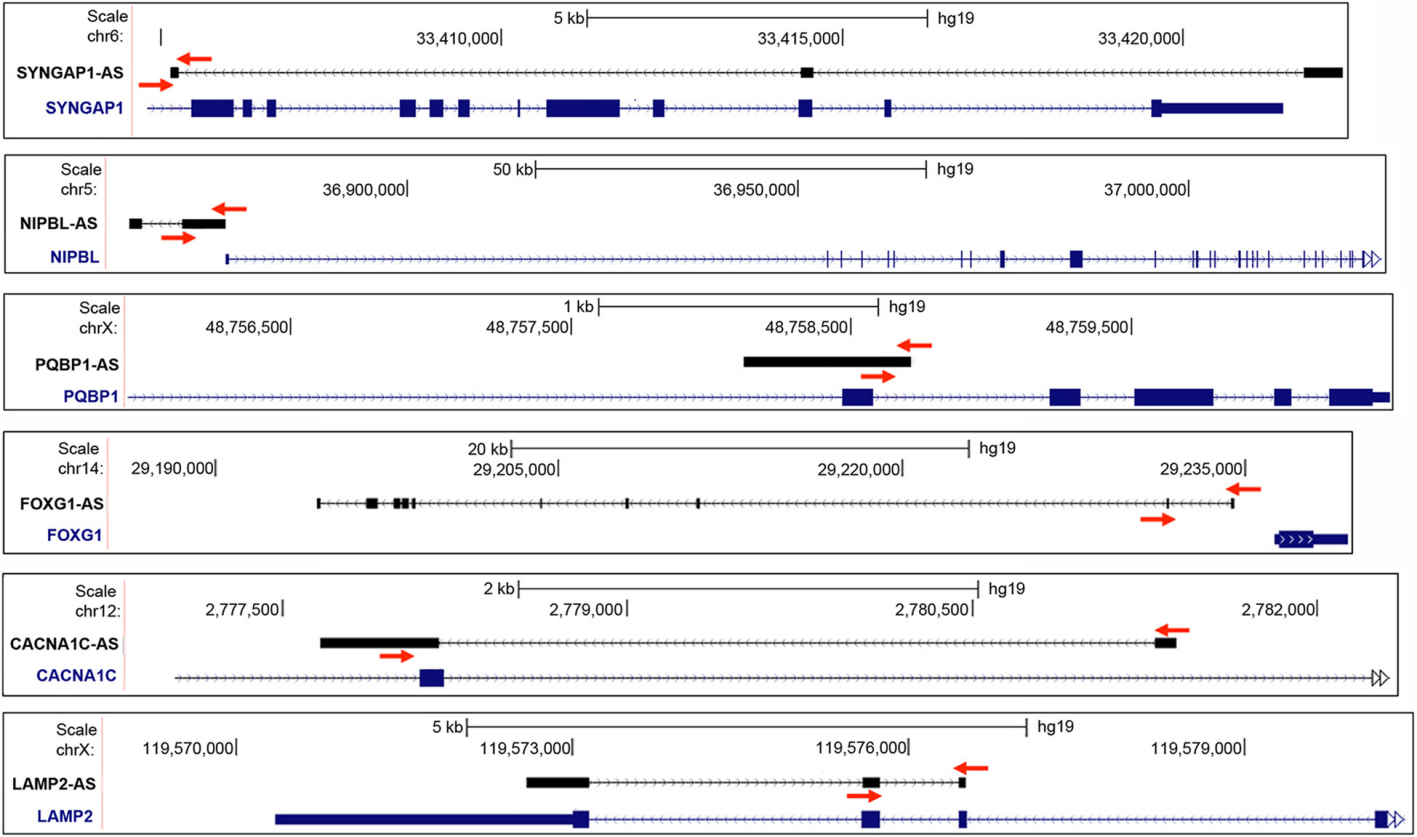
**Schematic representation of antisense and sense RNA partners.** Diagram showing the genomic location of antisense (in blue) and sense (in black) RNA pairs. The primers used to measure antisense RNAs expression by qRT-PCR are shown as red arrows.

Here, we investigated the expression patterns of ASD-NATs in the 9 PFC, 9 STG and 7 cerebella of non-ASD young individuals with average age of 15.79 ± 4.05 years (Additional file 1: Table S2). Among the 11 selected NATs, we found 6 to be differentially expressed within the examined brain regions. The structure, position in respect to the sense gene and location of the primers for these transcripts is depicted in Figure 3. Three of these transcripts, *SYNGAP1*-AS, *CACNA1C*-AS and *NIBPL*-AS have higher expression levels in the cerebellum as compared to the PFC and STG, while *PQBP1*-AS is more abundantly expressed in the PFC compared to both the STG and cerebellum and *LAMP2-AS* was expressed at a higher level in PFC compared to cerebellum (Figure 2a). The region-specific expression of the above antisense transcripts suggests a possible role in the development and function of the PFC, STG and cerebellum. The region-specificity of these transcripts suggests that NATs are not a product of random spurious transcription and provides a basis for future therapeutic approaches that could be tailored to specific regions of the brain, targeting non-protein-coding antisense targets instead of protein-coding genes.

### ASD-NATs show characteristic patterns of expressions with respect to their sense protein-coding partner

NATs can exert regulatory functions *in cis* by modulating the expression of neighboring genes [7,19,21]. In order to determine if ASD-NATs modulate the expression levels of protein-coding ASD-related genes through *cis*-regulation, we examined their respective expression levels. We observed discordant patterns of expression for two sense-antisense pairs: *SYNGAP1/SYNGAP1*-AS and *PQBP1/PQBP1*-AS. *SYNGAP1* was more highly expressed in the cortex compared to the cerebellum, whereas *PQBP1* is more abundant in the cerebellum (Figure 2b). Discordant expression of these exonic sense/antisense pairs suggests possible regulation of the protein-coding gene by its noncoding counterpart, a phenomenon already described for other loci [15,19,40]. Two other protein-coding genes, *NIPBL* and *FOXG1*, showed a pattern of region-specific expression similar to their promoter-associated antisense partners (Figure 2b). These NATs may have a positive regulatory effect on the sense partner [15], or the sense-antisense pairs might be co-regulated [41]. It is noteworthy that these two ASD-NATs overlap the promoter of their protein-coding sense partner, thus potentially sharing the same regulatory elements.

Overall, our data show that ASD-NATs show a regional expression pattern in the brain and further show discordant or concordant expression with regards to their sense partners, suggesting these noncoding antisense transcripts may perform highly specialized region-specific functions by affecting the expression of their sense partners.

### *SYNGAP1* antisense RNA (*SYNGAP1*-AS) is differentially expressed in ASD brain tissues compared to age-matched controls

The differential expression of ASD-NATs observed across brain regions suggests a tissue-specific function for these RNA transcripts. Thus, we hypothesized that expression of ASD-NATs may be altered in the brain of patients with autism compared to non-ASD cases. To test this hypothesis, we used qRT-PCR to measure the expression of the 10 ASD-NATs that we could detect in the PFC and STG of 18 (9 autistic and 9 age-matched individuals) and in the cerebella of 13 (7 autistic and 6 age-matched individuals).

**Figure 4:**
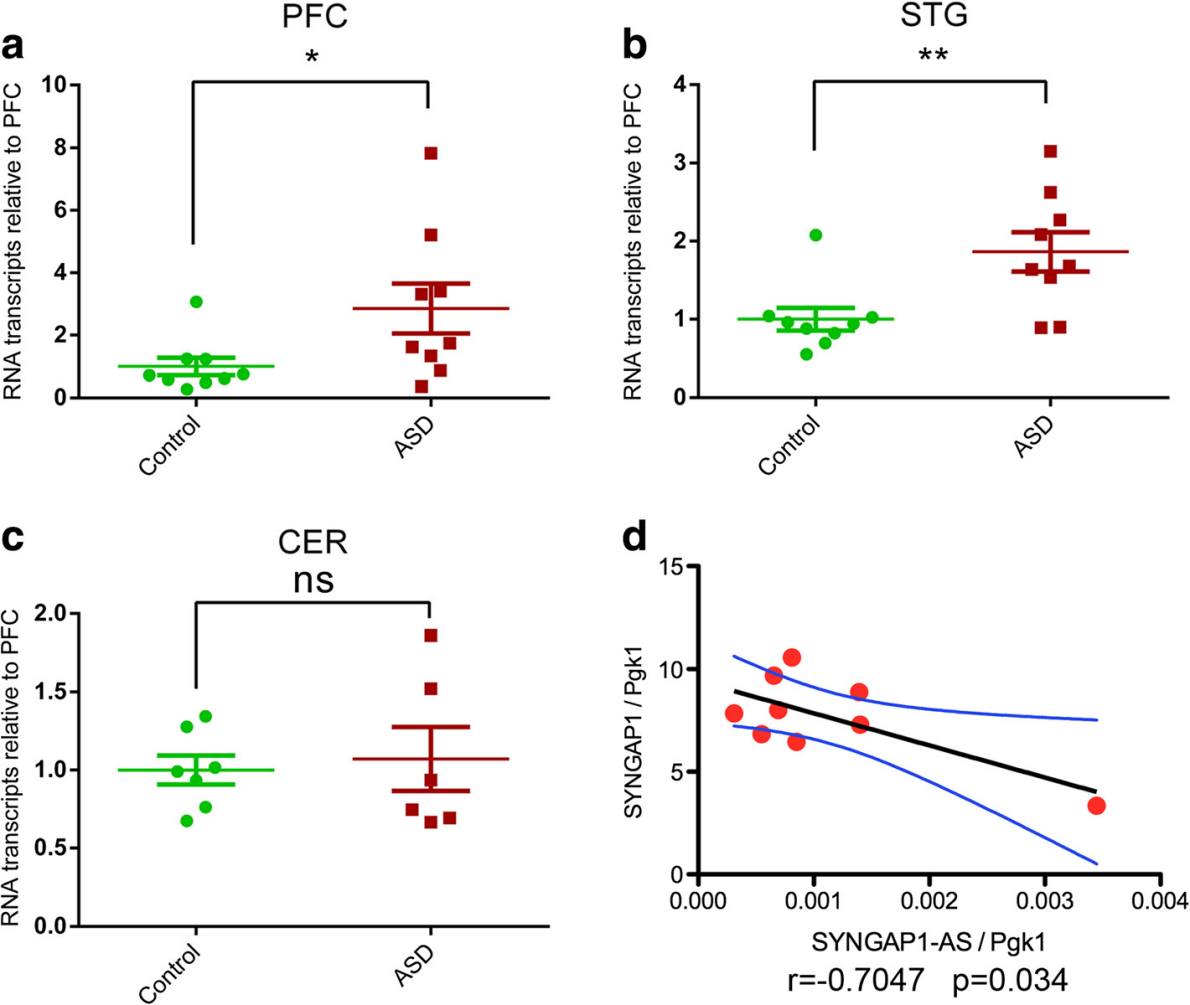
**Differential expression of *SYNGAP1*-AS in the postmortem brain of patients affected by ASD compared to age-matched controls.** Strand-specific qRT-PCR analysis of *SYNGAP1*-AS expression in: **(a)** Prefrontal cortex (PFC, n = 9), **(b)** Superior temporal gyrus (STG, n = 9) and **(c)** Cerebellum (CER, n = 7) of ASD patients and age-matched non-ASD individuals. *P <0.05, **P <0.01, Student’s *t*-test. **(d)** Linear regression plot demonstrating negative correlation of *SYNGAP1-AS* with *SYNGAP1* gene expression in the prefrontal cortex of control individuals. R = −0.7047*, p* <0.05, Pearson Correlation. *SYNGAP1*-AS expression is normalized to *PGK1*.

We found *SYNGAP1*-AS to be significantly upregulated (*p<*0.05) in the PFC and STG of autistic patients (Figure 4a and b), but not in the cerebellum (Figure 4c). The *SYNGAP1* gene codes for Synaptic Ras GTPase activating protein 1, which is critical for synapse function and is involved in cognition [42]. *SYNGAP1* plays a role in brain development as well as higher-order brain function, as mutations in this gene lead to mental retardation [43-45]. Although not statistically significant, three other ASD-NATs (*FOXG1-AS, VSP13B-AS, NHS-AS*) show an appreciable trend of differential expression in ASD (Additional file 7: Figure S4). Additionally, we found that the expression of *SYNGAP1*-AS negatively correlates with the expression of *SYNGAP1* sense gene in the prefrontal cortex of non-ASD individuals (Figure 4d). Our finding that *SYNGAP1*-AS expression is affected only in the PFC and STG and not in the cerebellum of autistic patients suggests that dysregulation of this non-protein-coding antisense transcript may be cortex-specific, leading to possible impairment of cortical function.

### Subcellular localization of ASD-NATs

Of the proposed functions of NATs [21], regulation of chromatin structure and epigenetic memory has received the most experimental support. Antisense transcripts have been shown to provide a scaffold by which proteins can interact with DNA and histones in a locus specific manner [19,46]. Thus, it is not surprising that ncRNAs are predominantly localized to the nucleus or associated with chromatin, while protein-coding RNAs are more abundant in the cytosol [16].

**Figure 5:**
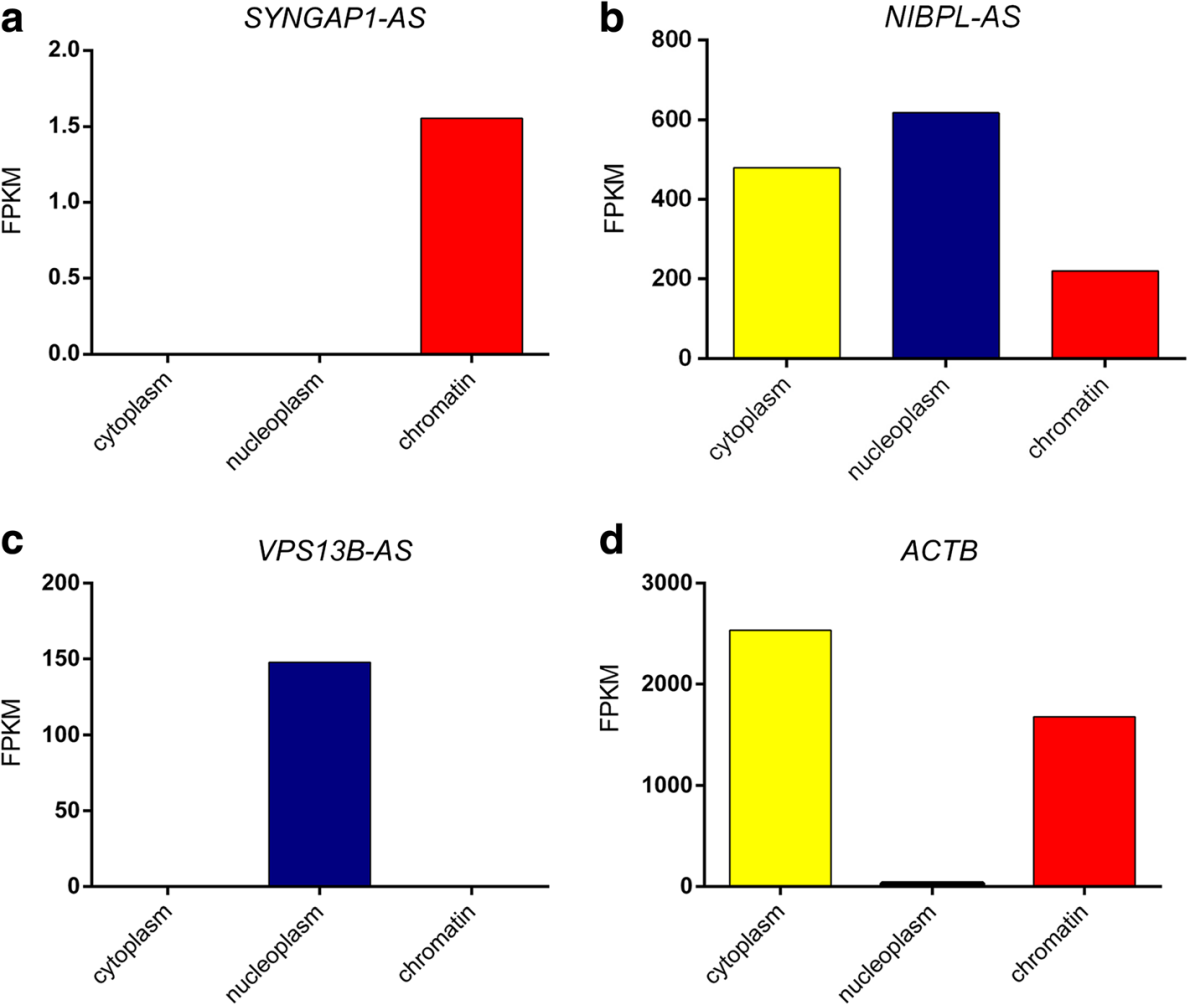
**Subcellular localization of antisense transcripts overlapping ASD-related genes.** RNAseq analysis of RNA extracted from the cytoplasm, nucleoplasm and chromatin of SH-SY5Y cells. Expression of *SYNGAP1*-AS **(a)**, *NIBPL*-AS **(b)**, *VPS13B*-AS **(c)**, and *β-actin* (ACTB) **(d)** is shown as fragments per kilobase of transcript per million reads mapped (FPKM).

In order to assess the subcellular localization of ASD-NATs, we isolated RNA from three cellular fractions (cytoplasm, nucleus, chromatin) of SH-SY5Y neuroblastoma cells and performed RNA sequencing (RNA-seq). FPKM reflecting expression levels of individual loci were used to further compare the expression of antisense RNAs between different compartments (Additional file 8: Dataset S1). We found that three out of 10 ASD-related NATs could be detected in SH-SY5Y cells using RNA-seq: *SYNGAP1*-AS, *VPS13B*-AS and *NIBPL*-AS. All three antisense transcripts were expressed predominantly in the nucleoplasm or chromatin compartments, while little or no expression was observed in the cytoplasm (Figure 5a-c). The pattern of subcellular localization of these ncRNAs is different from that of protein-coding genes such as beta-actin, which is largely localized to the cytoplasm (Figure 5d). The nuclear localization of these NATs offers evidence for the function of these transcripts in nuclear-associated processes and suggests that ASD-NATs might play a role in chromatin modifications or in transcriptional regulation. Overall, these data suggest that ASD-antisense RNAs overlapping genes previously implicated in ASD represent functional elements that may regulate brain function and development by regulating transcription of other genes.

## Discussion

Despite overwhelming evidence for the genetic causes of ASD, an exact mode of inheritance has not been elucidated and the wide phenotypic variability of ASD likely reflects the disruption of multiple gene networks and complex regulatory circuits within the genome. Recent data indicate that multiple genomic loci and several rare and highly penetrant gene variants (e.g., *NLGN3, NLGN4, SLC9A9, NRXN1, RPL10, SHANK2, SHANK3, CNTNAP2, PTCHD1*, and *PTEN* among others) are involved in ASD [5,6,47]. These genes and loci may account for 20–25% of children with ASD, but none of them can individually explain more than 2% of the cases [48].

Most eukaryotic genomes are transcribed as ncRNAs of various sizes ranging from 20 nucleotides to over 100 kb [16]. The number of ncRNAs in eukaryotic genomes increases as a function of developmental complexity [17,49,50]. Furthermore, many ncRNAs are expressed in the nervous system where they are thought to mediate fundamental biological functions [51,52]. Natural antisense transcripts have been reported for greater than 70% of transcriptional units within the human genome [17] and include primate-specific or human-specific [53] as well as other evolutionary conserved transcripts [54]. Aberrant expression of regulatory antisense RNAs might have defined consequences on the expression and/or function of protein-coding transcripts [15] and in some cases on the epigenetic status of the entire genomic loci [40,55]. Transcriptomic as well as *in vivo* studies have revealed the importance of several long ncRNAs in the maturation of neuronal cell subtypes [38,56,57]. These recent findings have raised the possibility of a more extensive role for long ncRNAs in regulating gene expression during neuronal differentiation and CNS development. Indeed, it was recently reported that several ncRNAs play functional roles in ASD. For example, a long ncRNA disrupted in schizophrenia 2 (*DISC2*) is a NAT overlapping the *DISC1* gene and has been implicated in schizophrenia, bipolar disorder [58] and autism [59]. A more recent report has indicated the presence of a non-protein-coding antisense RNA corresponding to suspected ASD locus at 5p14.1 [60]. This antisense RNA was shown to be strongly increased in post mortem brain tissue of ASD patients compared to control individuals and mechanistic studies suggested its role in regulating the level of the MOESIN protein. Moreover, mutations in an X-chromosome gene *PTCHD1* (*Patched Domain Containing 1*) were reported in several families with ASD and intellectual disability. Interestingly, deletion of 5’-flanking region of the gene containing a non-coding RNA were detected in several males with ASD while not present in controls [61]. Non-protein-coding antisense transcripts are reported in the Fragile X Mental Retardation gene (*FMR1*) locus. Fragile X syndrome (FXS) is the leading genetic cause of autism and intellectual disability among boys [62]. Although FXS is considered a monogenic disorder, there is evidence that supports an alternative model in which other ncRNAs contribute to FXS pathogenesis and to the observed phenotypic variations among patients [7,63]. We previously reported a ncRNA transcribed from the *FMR1* locus, *FMR4*, that is a 2.4 kb long, primate-specific transcript residing upstream of *FMR1* and which may have an anti-apoptotic function [7]. Therefore, the abundance of RNA produced by transcriptional events from nearly every region of the genome combined with the enrichment of ncRNA transcripts in the central nervous system make regulatory RNAs a prime target for mechanistic studies of neurodevelopmental disorders.

In the current study, we explored and validated the expression of ncRNAs in several reported ASD-related genomic loci utilizing bioinformatics and molecular biology approaches. Our bioinformatics pipeline allowed us to identify 71 noncoding antisense RNAs that overlap 38 of 103 genes previously implicated in ASD. These findings indicate that a large proportion of genomic loci implicated in ASD have a complex structure with transcription arising from both the plus and minus strands of DNA. Antisense transcripts can exert regulatory roles on gene expression in *cis* and *trans* and can be affected by mutations. Knockdown or blockade of endogenous antisense transcripts can have multiple outcomes, with the corresponding sense transcript concentration showing either an increase (discordant regulation) or a decrease (concordant regulation). It has been proposed that discordant de-repression of sense transcript expression, resulting in upregulation of sense RNA expression, can be achieved by removal or steric blockade of many but not all antisense transcripts. Here, we noticed that two exonic antisense RNAs, *SYNGAP1*-AS and *PQBP1*-AS, have tissue expression patterns that are discordant to that of their protein-coding partners, whereas two other promoter-associated antisense RNAs, *NIBPL*-AS and *FOXG1*-AS, have concordant tissue expression patterns with their sense genes. These findings suggest a possible functional regulation exerted by these antisense RNAs on their sense counterparts, a phenomenon already described for a subset of sense-antisense pairs [21]. Discordant pairs might interfere with transcription initiation from opposite strand, alter epigenetic structure of or may form double-stranded RNAs. Concordant pairs may potentially share the same regulatory elements, alter stability of sense mRNA or the sense-antisense transcripts are co-regulated as recently described for the majority of divergently transcribed long ncRNA/mRNA gene pairs, expressed during embryonic stem cell differentiation [41]. Thus, the presence of these transcripts in several ASD candidate genes suggests complex genomic structure of these loci and warrants functional studies that include both protein-coding genes and regulatory long noncoding antisense transcripts.

We demonstrated that 12 of the 18 randomly selected antisense RNAs overlapping ASD-NATs are expressed in the human brain where they can have specific regional expression, suggesting a possible region-specific function of these RNAs. Differential expression analysis of NATs in the PFC, STG and cerebellum revealed a significant increase in *SYNGAP1*-AS expression in the PFC and STG of autistic patients compared to control individuals. We also observed a statistically significant negative correlation of *SYNGAP1*-AS and *SYNGAP1* expression in the PFC of non-ASD individuals and a similar trend in the PFC of ASD patients. These data, together with the observed discordant regulation of *SYNGAP1*-AS and *SYNGAP1* mRNA, suggest a possible scenario in which upregulation of antisense RNAs lead to the dysregulation of the protein-coding gene expression.

Many noncoding RNAs function at the chromatin level, acting as scaffolds for the recruitment of functionally related epigenetic enzymes to specific loci [35,64-66]. The expression of these ncRNAs is usually restricted at the nuclear and chromatin level where they exert their function. RNA sequencing analysis of RNA expression in the cytoplasm, nucleoplasm and chromatin of the SH-SY5Y neuroblastoma cell line showed that some ASD-NATs have clear localization in the nucleoplasm or chromatin. The peculiar subcellular localization of these antisense RNAs implies that they may have functional roles in the nucleus and additionally supports the functionality of these ncRNAs in the cell. Among the chromatin-associated antisense RNAs, we found *SYNGAP1*-AS, providing additional support to the hypothesis that this NAT might have a regulatory function on its sense mRNA partner by mediating the epigenetic modifications of the regulatory elements controlling *SYNGAP1* expression.

## Conclusions

The data presented here provide strong evidence that the molecular network underlying ASD pathology is far more complex than anticipated and may involve dysregulation of ncRNAs. These regulatory elements, which are mostly ignored from current ASD genetics and functional studies, must be taken into account in order to obtain a more holistic view of the interplay of factors that lead to the disease state. We initiated a comprehensive genomic study of ASD that is not dependent solely on protein coding genes, and we demonstrate the expression of NATs in ASD-related genomic loci. Abundant transcriptions of regulatory ncRNAs in ASD-related genomic regions indicate that, in addition to conventional protein coding genes, disruption of RNA regulatory elements may contribute to the pathogenesis of ASD. Identification of disease specific RNAs [15], as well as novel technologies that enable targeting of these regulatory RNA molecules [40], adds a new dimension to current efforts investigating novel therapeutic targets for ASD.

## Abbreviations

ADI-R: Autism diagnostic interview revised; ASD: Autism spectrum disorders; FPKM: Fragments per kilobase of transcript per million reads mapped; NATs: Natural antisense transcripts; ncRNAs: Non-protein-coding RNAs.

## Competing interests

The authors declare that they have no competing interests.

## Authors’ contributions

DV developed bioinformatics pipeline, performed data mining, qRT-PCR, RNA-seq analysis, statistical analysis, prepared figures and drafted the manuscript. MM processed clinical samples, performed RNA extraction, helped in coordination of the project and drafted the manuscript. MAF conceived of the study, helped in its design and coordination, drafted the manuscript and performed RNA-sequencing. All authors read and approved the final manuscript.

## Supplementary Material

Additional file 1: Table S2 Patients informations. Click here for file

Additional file 2: Table S3 Primer sequences for qRT-PCR studies. Click here for file

Additional file 3: Figure S1 Schematic representation of Antisense and Sense RNA partners. Diagram showing the genomic location of Antisense (in blue) and Sense (in black) RNA partners. The primers used to measure antisense RNAs expression by qRT-PCR are shown as red arrows. Click here for file

Additional file 4 **Former appendix1.** Melting temperatures. Click here for file

Additional file 5: Table S1 Antisense RNAs to ASD-related genes. Click here for file

Additional file 6: Figure S3 ASD-related NATs expression in different human brain regions. qRT-PCR analysis of ASD-related NATs in the prefrontal cortex (PFC), superior temporal gyrus (STG) and cerebellum of non-ASD human postmortem brain samples. Transcripts expression is normalized to *PGK1*. Strand-specific qRT-PCR was used to measure expression of *ZNF81*-AS and *NHS*-AS. Click here for file

Additional file 7: Figure S4 Expression of *FOXG1-AS, VPS13B-AS* and *NHS-AS* in the non-ASD brain and in the brain of patients affected by ASD. qRT-PCR analysis of antisense RNAs expression in the non-ASD brain and brain affected by ASD pathology. (**a**) Expression of *FOXG1*-AS in the prefrontal cortex (PFC), (**b**) Expression of *VPS13B*-AS in the PFC and (**c**) Strand-specific qPCR analysis of *NHS*-AS in the superior temporal gyrus (STG). Antisense RNA expression is normalized to *PGK1. P* value – Student’s *t*-test. Click here for file

Additional file 8 **Dataset S1.** Gene expression in the cytoplasm, nucleoplasm and chromatin of SH-SY5Y cells based on RNA-seq. Click here for file

## References

[B1] Mefford HC Batshaw ML Hoffman EP Genomics, intellectual disability, and autism N Engl J Med 2012 366 733 743 22356326 10.1056/NEJMra1114194 PMC4107681

[B2] Muhle R Trentacoste SV Rapin I The genetics of autism Pediatrics 2004 113 e472 e486 15121991 10.1542/peds.113.5.e472

[B3] Anney R Klei L Pinto D Almeida J Bacchelli E Baird G Bolshakova N Bolte S Bolton PF Bourgeron T Brennan S Brian J Casey J Conroy J Correia C Corsello C Crawford EL de Jonge M Delorme R Duketis E Duque F Estes A Farrar P Fernandez BA Folstein SE Fombonne E Gilbert J Gillberg C Glessner JT Green A Individual common variants exert weak effects on the risk for autism spectrum disorderspi Hum Mol Genet 2012 21 4781 4792 22843504 10.1093/hmg/dds301 PMC3471395

[B4] Neale BM Kou Y Liu L Ma'ayan A Samocha KE Sabo A Lin CF Stevens C Wang LS Makarov V Polak P Yoon S Maguire J Crawford EL Campbell NG Geller ET Valladares O Schafer C Liu H Zhao T Cai G Lihm J Dannenfelser R Jabado O Peralta Z Nagaswamy U Muzny D Reid JG Newsham I Wu Y Patterns and rates of exonic de novo mutations in autism spectrum disorders Nature 2012 485 242 245 22495311 10.1038/nature11011 PMC3613847

[B5] Betancur C Etiological heterogeneity in autism spectrum disorders: more than 100 genetic and genomic disorders and still counting Brain Res 2011 1380 42 77 21129364 10.1016/j.brainres.2010.11.078

[B6] Miles JH Autism spectrum disorders--a genetics review Genet Med 2011 13 278 294 21358411 10.1097/GIM.0b013e3181ff67ba

[B7] Khalil AM Faghihi MA Modarresi F Brothers SP Wahlestedt C A novel RNA transcript with antiapoptotic function is silenced in fragile x syndrome PLoS ONE 2008 3 e1486 18213394 10.1371/journal.pone.0001486 PMC2194623

[B8] Faghihi MA Zhang M Huang J Modarresi F Van der Brug MP Nalls MA Cookson MR St-Laurent G 3rd Wahlestedt C Evidence for natural antisense transcript-mediated inhibition of microRNA function Genome Biol 2010 11 5 R56 20507594 10.1186/gb-2010-11-5-r56 PMC2898074

[B9] Scheele C Petrovic N Faghihi MA Lassmann T Fredriksson K Rooyackers O Wahlestedt C Good L Timmons JA The human PINK1 locus is regulated in vivo by a non-coding natural antisense RNA during modulation of mitochondrial function BMC Genomics 2007 8 74 17362513 10.1186/1471-2164-8-74 PMC1831481

[B10] Pastori C Wahlestedt C Involvement of long noncoding RNAs in diseases affecting the central nervous system RNA Biol 2012 9 860 870 22699553 10.4161/rna.20482 PMC3495748

[B11] Mercer TR Dinger ME Mariani J Kosik KS Mehler MF Mattick JS Noncoding RNAs in Long-Term Memory Formation Neuroscientist 2008 14 434 445 18997122 10.1177/1073858408319187

[B12] Pollard KS Salama SR Lambert N Lambot MA Coppens S Pedersen JS Katzman S King B Onodera C Siepel A Kern AD Dehay C Igel H Ares M Jr Vanderhaeghen P Haussler D An RNA gene expressed during cortical development evolved rapidly in humans Nature 2006 443 167 172 16915236 10.1038/nature05113

[B13] Mehler MF Mattick JS Non-coding RNAs in the nervous system J Physiol 2006 575 333 341 16809366 10.1113/jphysiol.2006.113191 PMC1819441

[B14] Qureshi IA Mattick JS Mehler MF Long non-coding RNAs in nervous system function and disease Brain Res 2010 1338 20 35 20380817 10.1016/j.brainres.2010.03.110 PMC2883659

[B15] Faghihi MA Modarresi F Khalil AM Wood DE Sahagan BG Morgan TE Finch CE St Laurent G 3rd Kenny PJ Wahlestedt C Expression of a noncoding RNA is elevated in Alzheimer's disease and drives rapid feed-forward regulation of beta-secretase Nat Med 2008 14 723 730 18587408 10.1038/nm1784 PMC2826895

[B16] Djebali S Davis CA Merkel A Dobin A Lassmann T Mortazavi A Tanzer A Lagarde J Lin W Schlesinger F Xue C Marinov GK Khatun J Williams BA Zaleski C Rozowsky J Röder M Kokocinski F Abdelhamid RF Alioto T Antoshechkin I Baer MT Bar NS Batut P Bell K Bell I Chakrabortty S Chen X Chrast J Curado J Landscape of transcription in human cells Nature 2012 489 101 108 22955620 10.1038/nature11233 PMC3684276

[B17] Carninci P Kasukawa T Katayama S Gough J Frith MC Maeda N Oyama R Ravasi T Lenhard B Wells C Kodzius R Shimokawa K Bajic VB Brenner SE Batalov S Forrest AR Zavolan M Davis MJ Wilming LG Aidinis V Allen JE Ambesi-Impiombato A Apweiler R Aturaliya RN Bailey TL Bansal M Baxter L Beisel KW Bersano T Bono H The transcriptional landscape of the mammalian genome Science 2005 309 1559 1563 16141072 10.1126/science.1112014

[B18] Katayama S Tomaru Y Kasukawa T Waki K Nakanishi M Nakamura M Nishida H Yap CC Suzuki M Kawai J Kodzius R Shimokawa K Bajic VB Brenner SE Batalov S Forrest AR Zavolan M Davis MJ Wilming LG Aidinis V Allen JE Ambesi-Impiombato A Apweiler R Aturaliya RN Bailey TL Bansal M Baxter L Beisel KW Bersano T Bono H Antisense transcription in the mammalian transcriptome Science 2005 309 1564 1566 16141073 10.1126/science.1112009

[B19] Magistri M Faghihi MA St Laurent G 3rd Wahlestedt C Regulation of chromatin structure by long noncoding RNAs: focus on natural antisense transcripts Trends Genet 2012 28 8 389 396 22541732 10.1016/j.tig.2012.03.013 PMC3768148

[B20] Wang KC Chang HY Molecular mechanisms of long noncoding RNAs Mol Cell 2011 43 904 914 21925379 10.1016/j.molcel.2011.08.018 PMC3199020

[B21] Faghihi MA Wahlestedt C Regulatory roles of natural antisense transcripts Nat Rev Mol Cell Biol 2009 10 637 643 19638999 10.1038/nrm2738 PMC2850559

[B22] Carthew RW Sontheimer EJ Origins and Mechanisms of miRNAs and siRNAs Cell 2009 136 642 655 19239886 10.1016/j.cell.2009.01.035 PMC2675692

[B23] Malone CD Hannon GJ Small RNAs as guardians of the genome Cell 2009 136 656 668 19239887 10.1016/j.cell.2009.01.045 PMC2792755

[B24] Chen J Sun M Hurst LD Carmichael GG Rowley JD Genome-wide analysis of coordinate expression and evolution of human cis-encoded sense-antisense transcripts Trends Genet 2005 21 326 329 15922830 10.1016/j.tig.2005.04.006

[B25] Trapnell C Pachter L Salzberg SL TopHat: discovering splice junctions with RNA-Seq Bioinformatics 2009 25 1105 1111 19289445 10.1093/bioinformatics/btp120 PMC2672628

[B26] Li H Handsaker B Wysoker A Fennell T Ruan J Homer N Marth G Abecasis G Durbin R The Sequence Alignment/Map format and SAMtools Bioinformatics 2009 25 2078 2079 19505943 10.1093/bioinformatics/btp352 PMC2723002

[B27] Trapnell C Williams BA Pertea G Mortazavi A Kwan G van Baren MJ Salzberg SL Wold BJ Pachter L Transcript assembly and quantification by RNA-Seq reveals unannotated transcripts and isoform switching during cell differentiation Nat Biotechnol 2010 28 511 515 20436464 10.1038/nbt.1621 PMC3146043

[B28] Thierry-Mieg D Thierry-Mieg J AceView: a comprehensive cDNA-supported gene and transcripts annotation Genome Biol 2006 7 Suppl 1 S12.1 14 16925834 10.1186/gb-2006-7-s1-s12 PMC1810549

[B29] Danesin C Houart C A Fox stops the Wnt: implications for forebrain development and diseases Curr Opin Genet Dev 2012 4 323 330 22742851 10.1016/j.gde.2012.05.001

[B30] Miyoshi G Fishell G Dynamic FoxG1 expression coordinates the integration of multipolar pyramidal neuron precursors into the cortical plate Neuron 2012 74 1045 1058 22726835 10.1016/j.neuron.2012.04.025 PMC3653132

[B31] Kortum F Das S Flindt M Morris-Rosendahl DJ Stefanova I Goldstein A Horn D Klopocki E Kluger G Martin P Rauch A Roumer A Saitta S Walsh LE Wieczorek D Uyanik G Kutsche K Dobyns WB The core FOXG1 syndrome phenotype consists of postnatal microcephaly, severe mental retardation, absent language, dyskinesia, and corpus callosum hypogenesis J Med Genet 2011 48 396 406 21441262 10.1136/jmg.2010.087528 PMC5522617

[B32] Kretz M Siprashvili Z Chu C Webster DE Zehnder A Qu K Lee CS Flockhart RJ Groff AF Chow J Johnston D Kim GE Spitale RC Flynn RA Zheng GX Aiyer S Raj A Rinn JL Chang HY Khavari PA Control of somatic tissue differentiation by the long non-coding RNA TINCR Nature 2013 493 231 235 23201690 10.1038/nature11661 PMC3674581

[B33] Sun L Goff LA Trapnell C Alexander R Lo KA Hacisuleyman E Sauvageau M Tazon-Vega B Kelley DR Hendrickson DG Yuan B Kellis M Lodish HF Rinn JL Long noncoding RNAs regulate adipogenesis Proc Natl Acad Sci USA 2013 110 3387 3392 23401553 10.1073/pnas.1222643110 PMC3587215

[B34] Kretz M Webster DE Flockhart RJ Lee CS Zehnder A Lopez-Pajares V Qu K Zheng GX Chow J Kim GE Rinn JL Chang HY Siprashvili Z Khavari PA Suppression of progenitor differentiation requires the long noncoding RNA ANCR Genes Dev 2012 26 338 343 22302877 10.1101/gad.182121.111 PMC3289881

[B35] Guttman M Donaghey J Carey BW Garber M Grenier JK Munson G Young G Lucas AB Ach R Bruhn L Yang X Amit I Meissner A Regev A Rinn JL Root DE Lander ES lincRNAs act in the circuitry controlling pluripotency and differentiation Nature 2011 477 295 300 21874018 10.1038/nature10398 PMC3175327

[B36] Klattenhoff CA Scheuermann JC Surface LE Bradley RK Fields PA Steinhauser ML Ding H Butty VL Torrey L Haas S Abo R Tabebordbar M Lee RT Burge CB Boyer LA Braveheart, a long noncoding RNA required for cardiovascular lineage commitment Cell 2013 152 570 583 23352431 10.1016/j.cell.2013.01.003 PMC3563769

[B37] Barry G Mattick JS The role of regulatory RNA in cognitive evolution Trends Cogn Sci 2012 16 497 503 22940578 10.1016/j.tics.2012.08.007

[B38] Mercer TR Qureshi IA Gokhan S Dinger ME Li G Mattick JS Mehler MF Long noncoding RNAs in neuronal-glial fate specification and oligodendrocyte lineage maturation BMC Neurosci 2010 11 14 20137068 10.1186/1471-2202-11-14 PMC2829031

[B39] Mercer TR Dinger ME Sunkin SM Mehler MF Mattick JS Specific expression of long noncoding RNAs in the mouse brain Proc Natl Acad Sci USA 2008 105 716 721 18184812 10.1073/pnas.0706729105 PMC2206602

[B40] Modarresi F Faghihi MA Lopez-Toledano MA Fatemi RP Magistri M Brothers SP van der Brug MP Wahlestedt C Inhibition of natural antisense transcripts in vivo results in gene-specific transcriptional upregulation Nat Biotechnol 2012 30 453 459 22446693 10.1038/nbt.2158 PMC4144683

[B41] Sigova AA Mullen AC Molinie B Gupta S Orlando DA Guenther MG Almada AE Lin C Sharp PA Giallourakis CC Young RA Divergent transcription of long noncoding RNA/mRNA gene pairs in embryonic stem cells Proc Natl Acad Sci USA 2013 110 2876 2881 23382218 10.1073/pnas.1221904110 PMC3581948

[B42] Kim JH Liao D Lau LF Huganir RL SynGAP: a synaptic RasGAP that associates with the PSD-95/SAP90 protein family Neuron 1998 20 683 691 9581761 10.1016/s0896-6273(00)81008-9

[B43] Hamdan FF Gauthier J Spiegelman D Noreau A Yang Y Pellerin S Dobrzeniecka S Cote M Perreau-Linck E Carmant L D'Anjou G Fombonne E Addington AM Rapoport JL Delisi LE Krebs MO Mouaffak F Joober R Mottron L Drapeau P Marineau C Lafrenière RG Lacaille JC Rouleau GA Michaud JL Synapse to Disease Group Mutations in SYNGAP1 in autosomal nonsyndromic mental retardation N Engl J Med 2009 360 599 605 19196676 10.1056/NEJMoa0805392 PMC2925262

[B44] Pinto D Pagnamenta AT Klei L Anney R Merico D Regan R Conroy J Magalhaes TR Correia C Abrahams BS Almeida J Bacchelli E Bader GD Bailey AJ Baird G Battaglia A Berney T Bolshakova N Bölte S Bolton PF Bourgeron T Brennan S Brian J Bryson SE Carson AR Casallo G Casey J Chung BH Cochrane L Corsello C Functional impact of global rare copy number variation in autism spectrum disorders Nature 2010 466 368 372 20531469 10.1038/nature09146 PMC3021798

[B45] Clement JP Aceti M Creson TK Ozkan ED Shi Y Reish NJ Almonte AG Miller BH Wiltgen BJ Miller CA Xu X Rumbaugh G Pathogenic SYNGAP1 mutations impair cognitive development by disrupting maturation of dendritic spine synapses Cell 2012 151 709 723 23141534 10.1016/j.cell.2012.08.045 PMC3500766

[B46] Rinn JL Chang HY Genome regulation by long noncoding RNAs Annu Rev Biochem 2012 81 145 166 22663078 10.1146/annurev-biochem-051410-092902 PMC3858397

[B47] O’Roak BJ Vives L Fu W Egertson JD Stanaway IB Phelps IG Carvill G Kumar A Lee C Ankenman K Munson J Hiatt JB Turner EH Levy R O’Day DR Krumm N Coe BP Martin BK Borenstein E Nickerson DA Mefford HC Doherty D Akey JM Bernier R Eichler EE Shendure J Multiplex targeted sequencing identifies recurrently mutated genes in autism spectrum disorders Science 2012 6114 1619 1622 23160955 10.1126/science.1227764 PMC3528801

[B48] Voineagu I Gene expression studies in autism: moving from the genome to the transcriptome and beyond Neurobiol Dis 2012 45 1 69 75 21839838 10.1016/j.nbd.2011.07.017

[B49] Liu G Mattick JS Taft RJ A meta-analysis of the genomic and transcriptomic composition of complex life Cell Cycle 2013 13 2061 2072 23759593 10.4161/cc.25134 PMC3737309

[B50] Kapranov P Cheng J Dike S Nix DA Duttagupta R Willingham AT Stadler PF Hertel J Hackermuller J Hofacker IL Bell I Cheung E Drenkow J Dumais E Patel S Helt G Ganesh M Ghosh S Piccolboni A Sementchenko V Tammana H Gingeras TR RNA maps reveal new RNA classes and a possible function for pervasive transcription Science 2007 316 1484 1488 17510325 10.1126/science.1138341

[B51] St Laurent G 3rd Wahlestedt C Noncoding RNAs: couplers of analog and digital information in nervous system function? Trends Neurosci 2007 30 612 621 17996312 10.1016/j.tins.2007.10.002

[B52] Ng SY Bogu GK Soh BS Stanton LW The Long Noncoding RNA RMST Interacts with SOX2 to regulate Neurogenesis Mol Cell 2013 3 349 359 23932716 10.1016/j.molcel.2013.07.017

[B53] Bush EC Lahn BT A genome-wide screen for noncoding elements important in primate evolution BMC Evol Biol 2008 8 17 18215302 10.1186/1471-2148-8-17 PMC2242780

[B54] Engstrom PG Suzuki H Ninomiya N Akalin A Sessa L Lavorgna G Brozzi A Luzi L Tan SL Yang L Kunarso G Ng EL Batalov S Wahlestedt C Kai C Kawai J Carninci P Hayashizaki Y Wells C Bajic VB Orlando V Reid JF Lenhard B Lipovich L Complex Loci in human and mouse genomes PLoS Genet 2006 2 e47 16683030 10.1371/journal.pgen.0020047 PMC1449890

[B55] Yap KL Li S Munoz-Cabello AM Raguz S Zeng L Mujtaba S Gil J Walsh MJ Zhou MM Molecular interplay of the noncoding RNA ANRIL and methylated histone H3 lysine 27 by polycomb CBX7 in transcriptional silencing of INK4a Mol Cell 2010 38 662 674 20541999 10.1016/j.molcel.2010.03.021 PMC2886305

[B56] Bond AM Vangompel MJ Sametsky EA Clark MF Savage JC Disterhoft JF Kohtz JD Balanced gene regulation by an embryonic brain ncRNA is critical for adult hippocampal GABA circuitry Nat Neurosci 2009 12 1020 1027 19620975 10.1038/nn.2371 PMC3203213

[B57] Qureshi IA Mehler MF Emerging roles of non-coding RNAs in brain evolution, development, plasticity and disease Nat Rev Neurosci 2012 13 528 541 22814587 10.1038/nrn3234 PMC3478095

[B58] Millar JK James R Brandon NJ Thomson PA DISC1 and DISC2: discovering and dissecting molecular mechanisms underlying psychiatric illness Ann Med 2004 36 367 378 15478311 10.1080/07853890410033603

[B59] Williams JM Beck TF Pearson DM Proud MB Cheung SW Scott DA A 1q42 deletion involving DISC1, DISC2, and TSNAX in an autism spectrum disorder Am J Med Genet A 2009 149A 1758 1762 19606485 10.1002/ajmg.a.32941 PMC2909829

[B60] Kerin T Ramanathan A Rivas K Grepo N Coetzee GA Campbell DB A noncoding RNA antisense to moesin at 5p14.1 in autism Sci Transl Med 2012 4 128ra140 10.1126/scitranslmed.3003479 22491950

[B61] Noor A Whibley A Marshall CR Gianakopoulos PJ Piton A Carson AR Orlic-Milacic M Lionel AC Sato D Pinto D Drmic I Noakes C Senman L Zhang X Mo R Gauthier J Crosbie J Pagnamenta AT Munson J Estes AM Fiebig A Franke A Schreiber S Stewart AF Roberts R McPherson R Guter SJ Cook EH Jr Dawson G Schellenberg GD Disruption at the PTCHD1 Locus on Xp22.11 in Autism spectrum disorder and intellectual disability Sci Transl Med 2010 2 3001267 10.1126/scitranslmed.3001267 PMC2987731 20844286

[B62] De Rubeis S Bagni C Regulation of molecular pathways in the Fragile X Syndrome: insights into Autism Spectrum Disorders J Neurodev Disord 2011 3 257 269 21842222 10.1007/s11689-011-9087-2 PMC3167042

[B63] Ladd PD Smith LE Rabaia NA Moore JM Georges SA Hansen RS Hagerman RJ Tassone F Tapscott SJ Filippova GN An antisense transcript spanning the CGG repeat region of FMR1 is upregulated in premutation carriers but silenced in full mutation individuals Hum Mol Genet 2007 16 3174 3187 17921506 10.1093/hmg/ddm293

[B64] Gupta RA Shah N Wang KC Kim J Horlings HM Wong DJ Tsai MC Hung T Argani P Rinn JL Wang Y Brzoska P Kong B Li R West RB van de Vijver MJ Sukumar S Chang HY Long non-coding RNA HOTAIR reprograms chromatin state to promote cancer metastasis Nature 2010 464 1071 1076 20393566 10.1038/nature08975 PMC3049919

[B65] Nagano T Mitchell JA Sanz LA Pauler FM Ferguson-Smith AC Feil R Fraser P The Air noncoding RNA epigenetically silences transcription by targeting G9a to chromatin Science 2008 322 1717 1720 18988810 10.1126/science.1163802

[B66] Wang KC Yang YW Liu B Sanyal A Corces-Zimmerman R Chen Y Lajoie BR Protacio A Flynn RA Gupta RA Wysocka J Lei M Dekker J Helms JA Chang HY A long noncoding RNA maintains active chromatin to coordinate homeotic gene expression Nature 2011 472 120 124 21423168 10.1038/nature09819 PMC3670758

