# Supplementary material for: Expression of non-protein-coding antisense RNAs in genomic regions related to autism spectrum disorders
Source: Mol Autism. 2013 Sep 4;4:32. doi: 10.1186/2040-2392-4-32 (PMC3851999; doi:10.1186/2040-2392-4-32)
Supplement: Additional file 1: Table S2 — Patients informations. [file 2040-2392-4-32-S1.doc]

**Table S2.** Patient information

| **ID** | **Disorder** | **Tissue Description** | **Cause of Death** | **Age of Death** | **Sex** | **Race** | **Post Mortem Interval (hours)** | **ADI-R** |
| --- | --- | --- | --- | --- | --- | --- | --- | --- |
| 5391 | Ctrl | PFC, STG, CER | Drowning | 8 years, 286 days | M | Caucasian | 12 |  |
| 914 | Ctrl | PFC, STG, CER | Accident | 20 years, 50 days | M | Caucasian | 18 |  |
| 1024 | Ctrl | PFC, STG | Cardiac Arrhythmia | 14 years, 60 days | M | Caucasian | 16 |  |
| 1158 | Ctrl | PFC, STG, CER | Cardiomegaly | 16 years, 63 days | M | Caucasian | 15 |  |
| 1347 | Ctrl | PFC, STG, CER | Multiple Injuries | 19 years, 76 days | F | Caucasian | 16 |  |
| 1846 | Ctrl | PFC, STG, CER | Multiple Injuries | 20 years, 221 days | F | Caucasian | 9 |  |
| 4669 | Ctrl | PFC, STG, CER | Neck and Head Injuries | 16 years, 125 days | M | Caucasian | 16 |  |
| 5168 | Ctrl | PFC, STG, CER | Cardiac Arrhythmia | 15 years, 361 days | F | Caucasian | 11 |  |
| 5173 | Ctrl | PFC, STG | Asthma | 10 years, 279 days | F | Caucasian | 10 |  |
| 5278 | ASD | PFC, STG, CER | Drowning | 15 years, 324 days | F | Caucasian | 13 | YES |
| 5302 | ASD | PFC, STG | Diabetic ketoacidosis | 16 years, 119 days | M | Caucasian | 20 | NO |
| 5308 | ASD | PFC, STG, CER | Skull fractures | 4 year, 182 days | M | Caucasian | 21 | YES |
| 5403 | ASD | PFC, STG, CER | Cardiac Arrhythmia | 16 years, 266 days | M | Caucasian | 35 | YES |
| 5419 | ASD | PFC, STG, CER | Natural | 19 years, 350 days | F | Caucasian | 22 | YES |
| 1638 | ASD | PFC, STG | Seizure Disorder | 20 years, 277 days | F | Caucasian | 50 | YES |
| 4899 | ASD | PFC, STG | Drowning | 14 years, 126 days | M | Caucasian | 9 | YES |
| 4999 | ASD | PFC, STG, CER | Cardiac Arrhythmia | 20 years, 274 days | M | Caucasian | 14 | NO |
| 5144 | ASD | PFC, STG, CER | Cancer | 7 years, 55 days | M | Caucasian | 3 | YES |
