# Supplementary material for: Expression of non-protein-coding antisense RNAs in genomic regions related to autism spectrum disorders
Source: Mol Autism. 2013 Sep 4;4:32. doi: 10.1186/2040-2392-4-32 (PMC3851999; doi:10.1186/2040-2392-4-32)
Supplement: Additional file 2: Table S3 — Primer sequences for qRT-PCR studies. [file 2040-2392-4-32-S2.doc]

**Table S3.** Primer sequences for qRT-PCR studies

| **NAME** | **FORWARD PRIMER** | **REVERSE PRIMER** |
| --- | --- | --- |
| AHI1-AS | TCAGCAGAGACCAGAAGGCTA | CGTGGTTGTCTGCAATCAGT |
| BRAF-AS | ATCTCCCACCTCAGCCTCTT | CACTTCCAAGTCATCCCAGAA |
| CACNA1C-AS | TGGTACTGGACTTGGGATGA | CGGGACAGTCATGTTCAATG |
| CACNA1C-AS2 | TCTTTCCAACCAGGCAGTTC | TGAACACGACGCTTAACCTCT |
| CNTNAP2-AS | TGATCAGCTGTGAAGAATTGG | GCTCCCTTGCAGAGAAAAGA |
| DHCR7-AS | CGCAAGGAGCAAAACAGAC | CAGTCTCTTGTCCAATCACAGC |
| FOXG1-AS | AGAGGGAGGTGGAGAAGCAT | ACCTGGATGCCTCTGTATGG |
| FOXP1-AS | TCCAAAGTGCTGGGATTACA | CGATTAGTTGGCATCCGTTT |
| LAMP2-AS | CATCCCAGTAGCTGAGATTGTT | TGAAGGAAGTGAACATCAGCA |
| MBD5-AS | CAACTTCAAGCATTGGCAGA | GATGGTGAGGGACACATTCA |
| NHS-AS | CACCAGAGACGTCGTCGTAA | CCCCTCTCCTCATTCCAAGT |
| NIBPL-AS | AGCTTTAGGGAGCCGATCTC | ACCATTTAAAACGGGCATCA |
| NRXN1-AS | ATCCCAACCTCTGTGTGCAT | TTGGGCCTGAGTTCTTTCAG |
| PQBP1-AS | CCTTGTGTCCCCCTAAACCT | GAGGGGGAGCTAACTTCTGG |
| PTEN-AS | AGCAAAAACTAGGTGCAAGGA | ATTGTGCATTTTGGCATCTG |
| SYNGAP1-AS | GCCAGCCATGGTATCTCATT | ACTCCTGACCTCAGGCAATC |
| VPS13B-AS | AGCACGTAAAGCCCTGAAAA | TGCTTCCGTTGTGATACTGC |
| ZNF81-AS | GTCATTGCTTCGCAGCTGTA | TCTTGGACTCTACCCCTCGT |
