# Supplementary figures and images for: Expression of non-protein-coding antisense RNAs in genomic regions related to autism spectrum disorders
Source: Mol Autism. 2013 Sep 4;4:32. doi: 10.1186/2040-2392-4-32 (PMC3851999; doi:10.1186/2040-2392-4-32)

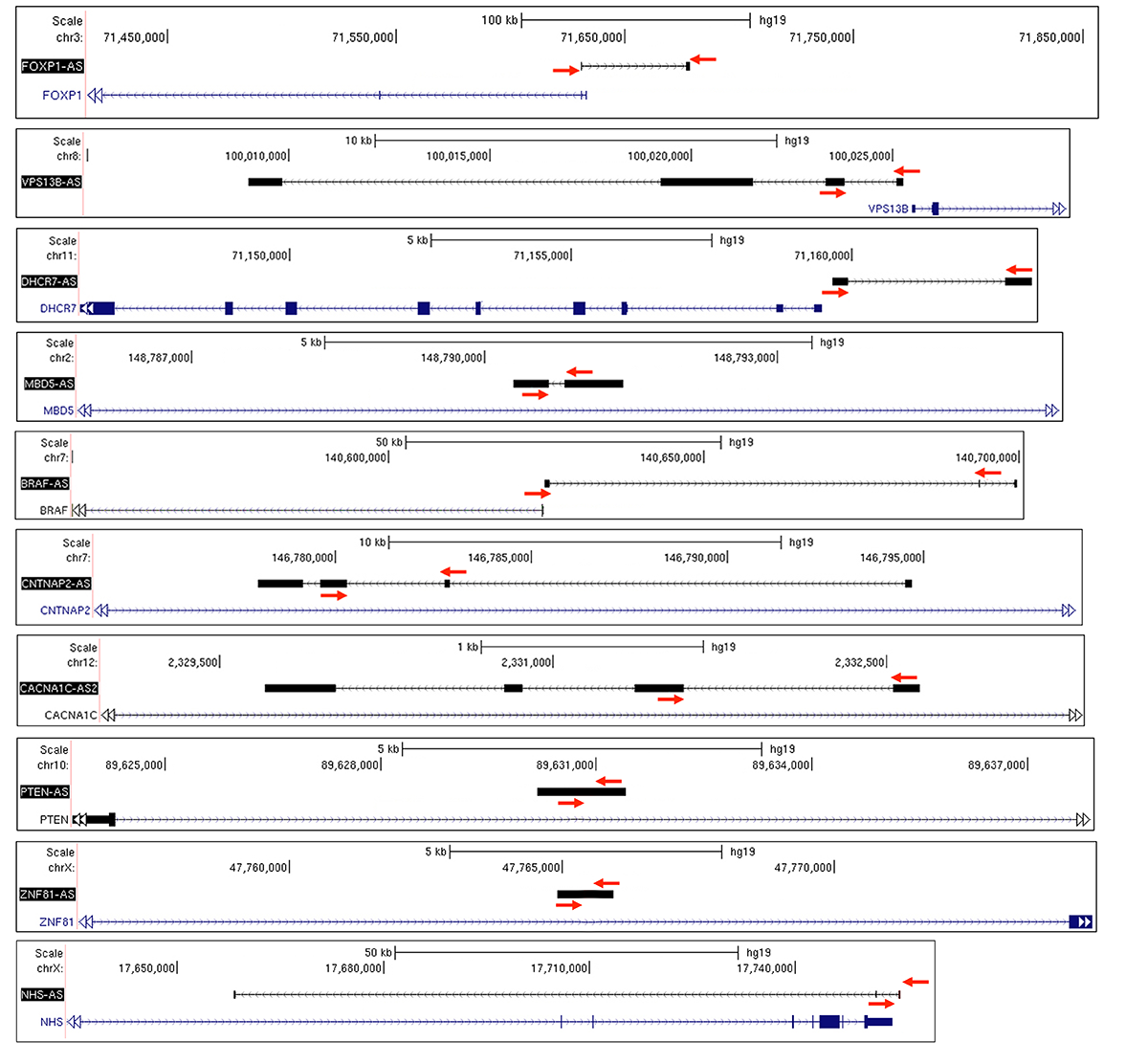

Supplement: Additional file 3: Figure S1 — Schematic representation of Antisense and Sense RNA partners. Diagram showing the genomic location of Antisense (in blue) and Sense (in black) RNA partners. The primers used to measure antisense RNAs expression by qRT-PCR are shown as red arrows. [file 2040-2392-4-32-S3.tiff]

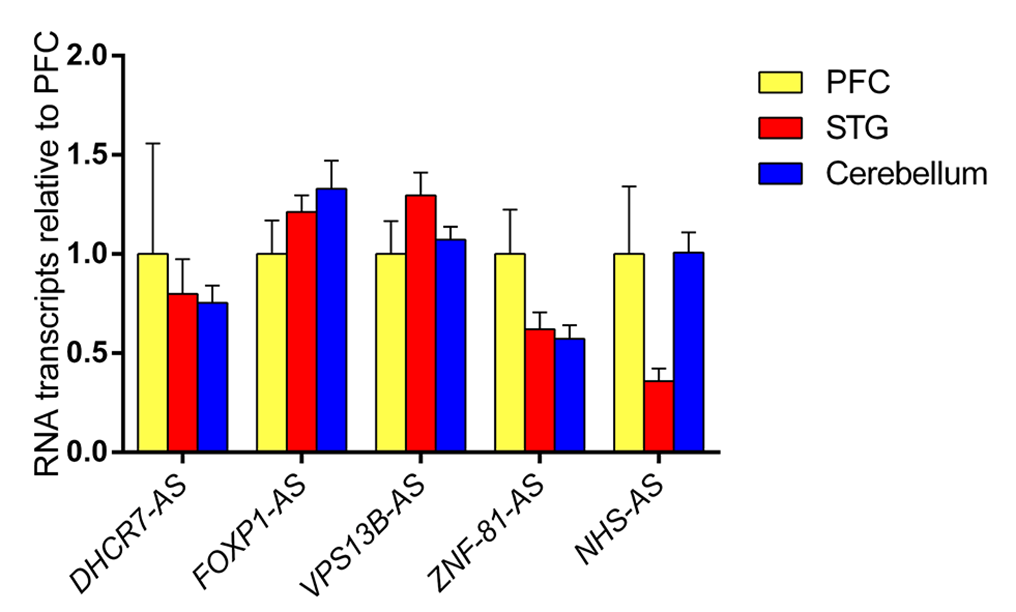

Supplement: Additional file 6: Figure S3 — ASD-related NATs expression in different human brain regions. qRT-PCR analysis of ASD-related NATs in the prefrontal cortex (PFC), superior temporal gyrus (STG) and cerebellum of non-ASD human postmortem brain samples. Transcripts expression is normalized to PGK1. Strand-specific qRT-PCR was used to measure expression of ZNF81-AS and NHS-AS. [file 2040-2392-4-32-S6.tiff]

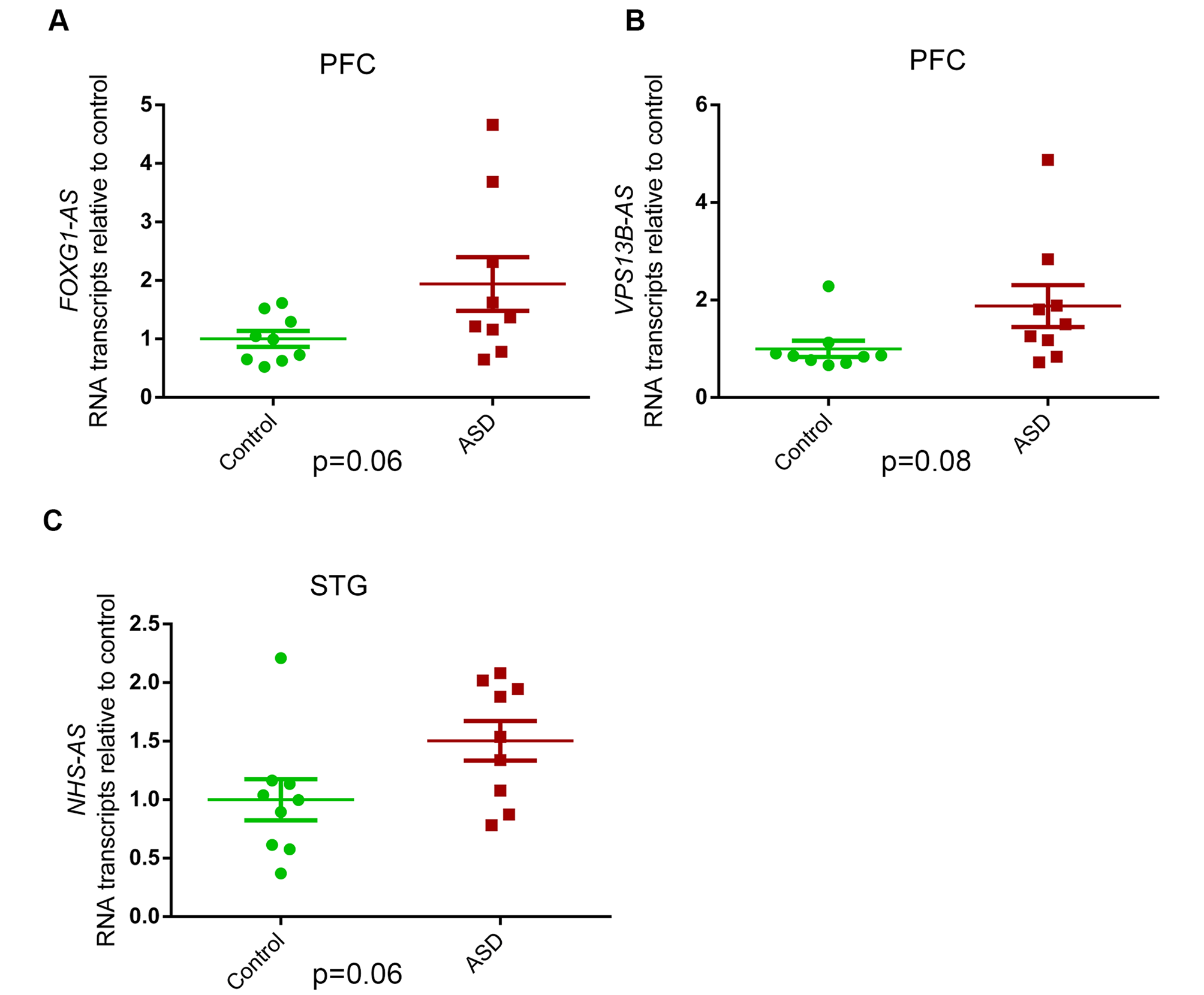

Supplement: Additional file 7: Figure S4 — Expression of FOXG1-AS, VPS13B-AS and NHS-AS in the non-ASD brain and in the brain of patients affected by ASD. qRT-PCR analysis of antisense RNAs expression in the non-ASD brain and brain affected by ASD pathology. (a) Expression of FOXG1-AS in the prefrontal cortex (PFC), (b) Expression of VPS13B-AS in the PFC and (c) Strand-specific qPCR analysis of NHS-AS in the superior temporal gyrus (STG). Antisense RNA expression is normalized to PGK1. P value – Student’s t-test. [file 2040-2392-4-32-S7.tiff]
