# Supplementary material for: Expression of non-protein-coding antisense RNAs in genomic regions related to autism spectrum disorders
Source: Mol Autism. 2013 Sep 4;4:32. doi: 10.1186/2040-2392-4-32 (PMC3851999; doi:10.1186/2040-2392-4-32)

BRAF-AS

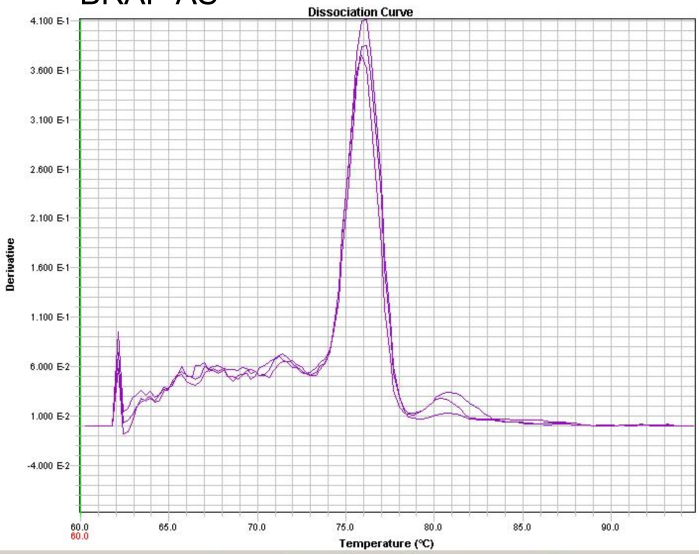

CACNA1C-AS

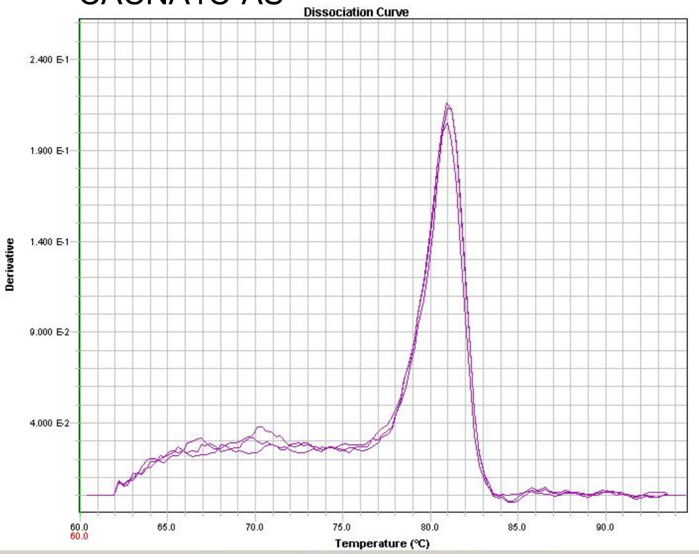

CACNA1C-AS2

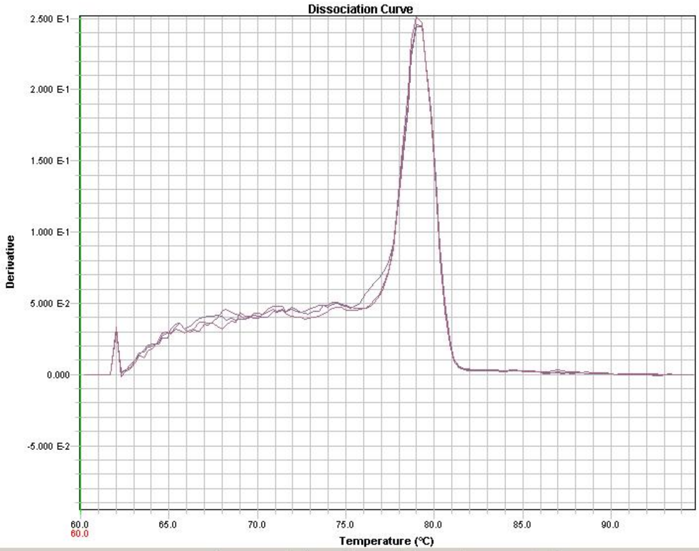

CNTNAP2-AS

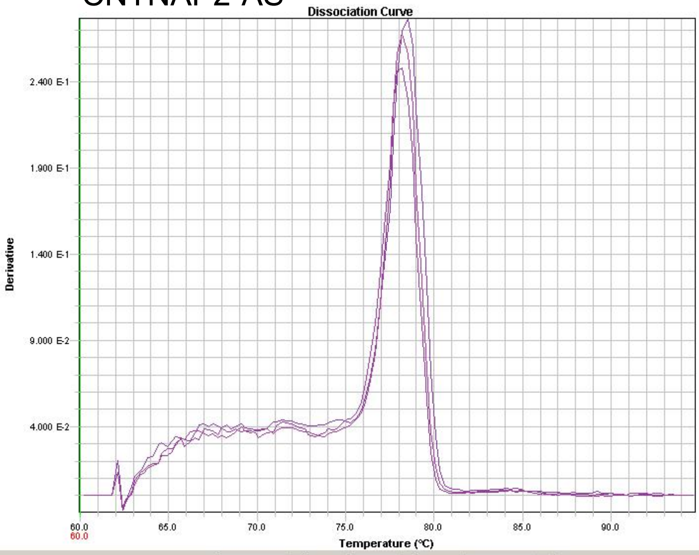

DHCR7-AS

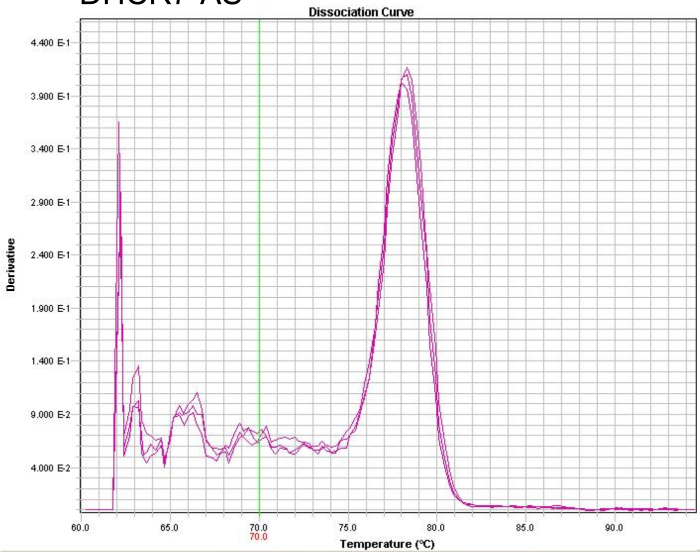

FOXG1-AS

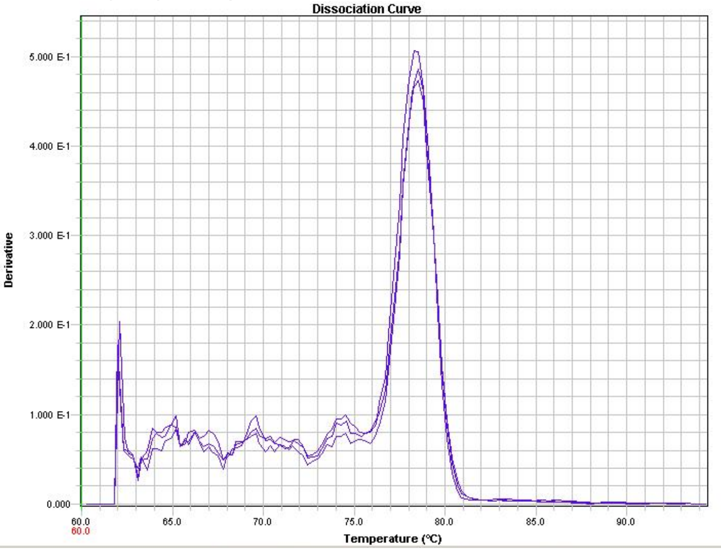

FOXP1-AS

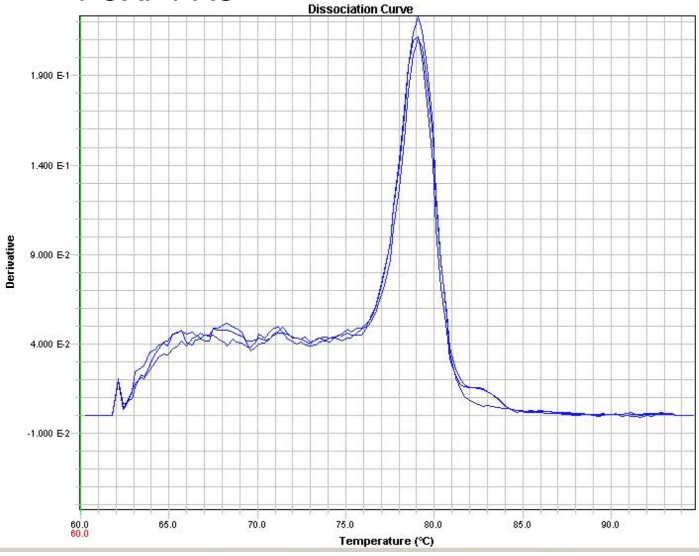

LAMP2-AS

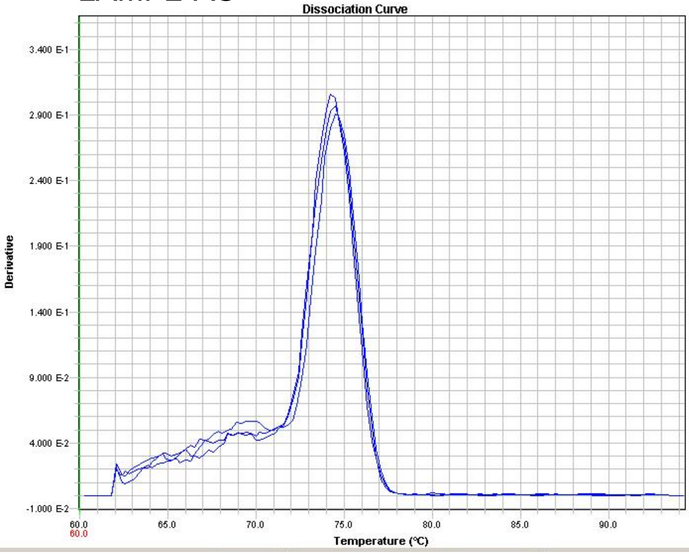

MDB5-AS

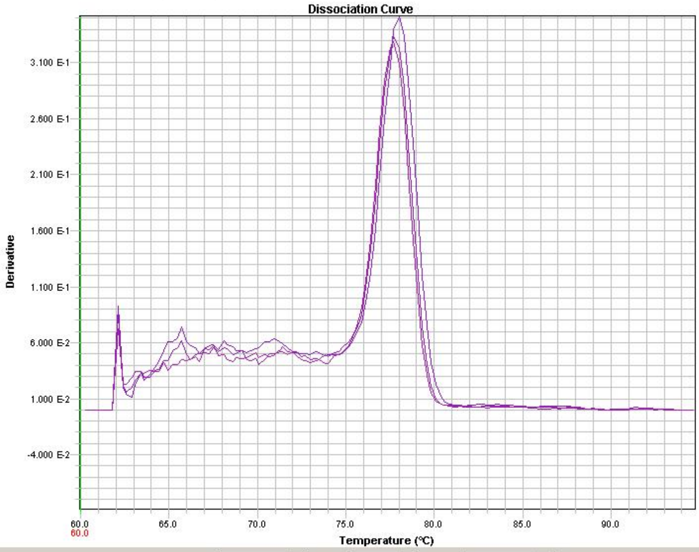

NHS-AS

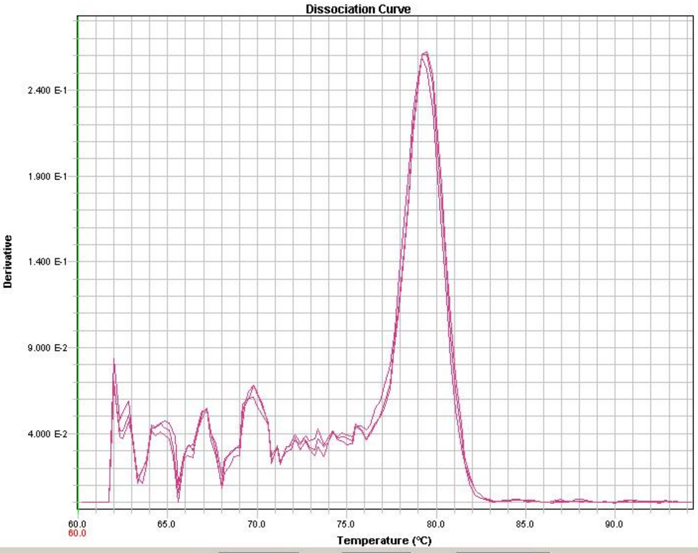

NIPBL-AS

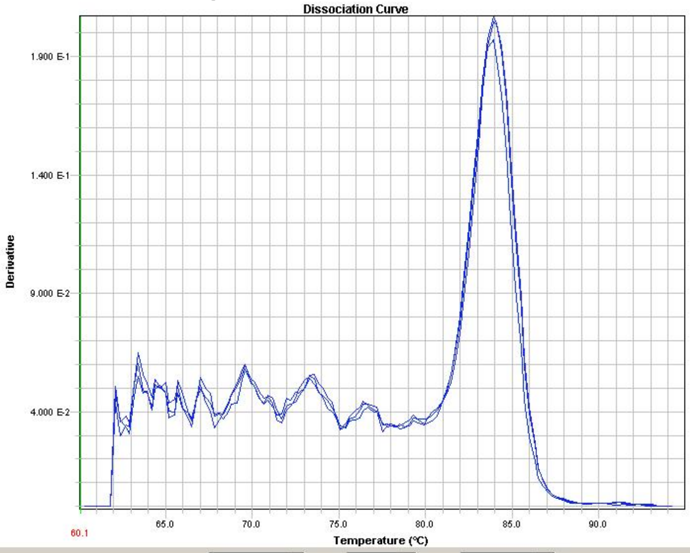

PQBP-AS

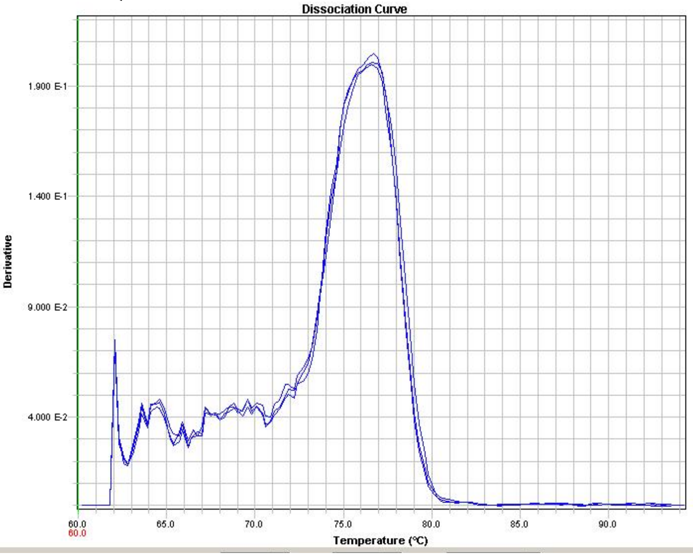

PTEN-AS

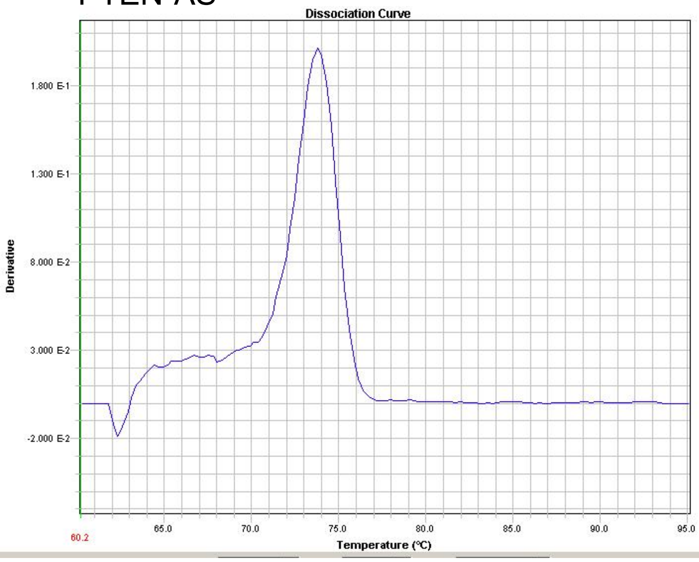

SYNGAP1-AS

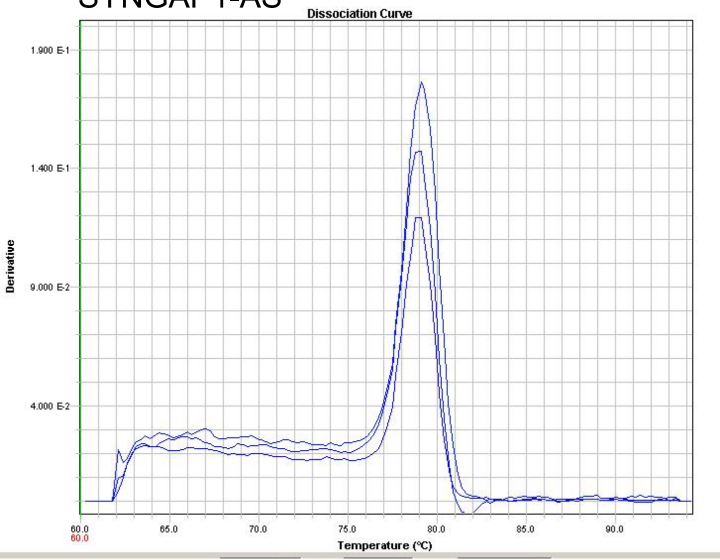

PTEN-AS

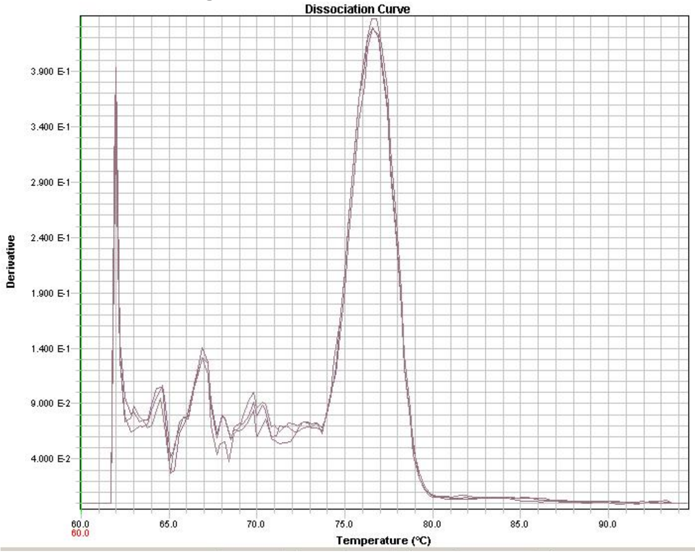

ZNF-AS

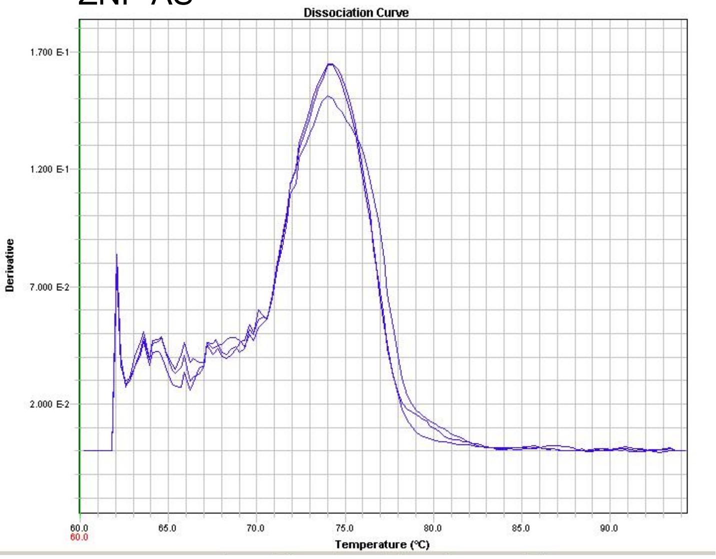

Supplement: Additional file 4 — Former appendix1. Melting temperatures. [file 2040-2392-4-32-S4.pdf]
