# Supplementary material for: Expression of non-protein-coding antisense RNAs in genomic regions related to autism spectrum disorders
Source: Mol Autism. 2013 Sep 4;4:32. doi: 10.1186/2040-2392-4-32 (PMC3851999; doi:10.1186/2040-2392-4-32)
Supplement: Additional file 5: Table S1 — Antisense RNAs to ASD-related genes. [file 2040-2392-4-32-S5.doc]

**Table S1. Antisense RNAs to ASD-relat**ed genes

| **Sense gene name** | **Antisense gene coordinates** | **Antisense AceView name** | **Antisense type** |
| --- | --- | --- | --- |
| NRXN1 | 2:50652867-50655736,1 | molar | intronic |
| NRXN1 | 2:50858398-50860142,1 | peedoy | intronic |
| NPHP1 | 2:110942719-110943081,1 | LOC100287074 | intronic |
| MBD5 | 2:148791418-148790296,-1 | starklee | intronic |
| SATB2 | 2:200190032-200194125,1 | seydu | exonic |
| SATB2 | 2:200209479-200209858,1 | stykly | intronic |
| FOXP1 | 3:71630795-71678203,1 | chyrarbu | exonic;promoter |
| FOXP1 | 3:71125048-71125996,1 | darubu | intronic |
| FOXP1 | 3:71628757-71629323,1 | flaglor | intronic |
| FOXP1 | 3:71123225-71127054,1 | nerswerby | exonic |
| FOXP1 | 3:71382412-71383549,1 | noyswerby | intronic;promoter |
| FOXP1 | 3:71338920-71400593,1 | paswerby | intronic;promoter |
| FOXP1 | 3:71122320-71124242,1 | swoyrybu | intronic |
| NIPBL | 5:36876787-36864527,-1 | LOC646719 | promoter |
| MEF2C | 5:87972036-88020761,1 | snostawby | exonic |
| NSD1 | 5:176692316-176691411,-1 | fafley | intronic;promoter |
| AHI1 | 6:135818939-136037193,1 | NCRNA00271 | promoter |
| AHI1 | 6:135635690-135636042,1 | peespoyby | intronic |
| AHI1 | 6:135645879-135649097,1 | plafla | intronic;promoter |
| AHI1 | 6:135622656-135628303,1 | skarsloyby | exonic;promoter |
| SYNGAP1 | 6:33422342-33405140,-1 | kleefloybu | exonic;promoter |
| BRAF | 7:140624758-140699636,1 | kablaw | promoter |
| BRAF | 7:140431596-140431900,1 | nawshyby | intronic |
| BRAF | 7:140497692-140498459,1 | peeshyby | intronic |
| CNTNAP2 | 7:146794706-146778028,-1 | deyko | intronic |
| CNTNAP2 | 7:148117213-148116049,-1 | glortorbo | exonic |
| CNTNAP2 | 7:146095845-146095363,-1 | vawshuby | intronic |
| CHD7 | 8:61722165-61721280,-1 | merly | exonic |
| VPS13B | 8:100884088-100882838,-1 | bladyby | exonic |
| VPS13B | 8:100811418-100808838,-1 | muvawby | intronic |
| VPS13B | 8:100106288-100103724,-1 | smergee | intronic |
| VPS13B | 8:100026175-100008986,-1 | speeshor | promoter |
| EHMT1 | 9:140730579-140729873,-1 | fawswey | exonic |
| PTEN | 10:89631419-89630176,-1 | kloloy | intronic |
| PTEN | 10:89664045-89663740,-1 | zarsny | intronic |
| DHCR7 | 11:71159652-71163207,1 | steymor | intronic;promoter |
| SHANK2 | 11:70486421-70493667,1 | spumor | intronic |
| SHANK2 | 11:70881635-70882211,1 | stumor | intronic |
| CACNA1C | 12:2800366-2785139,-1 | bleetybo | exonic |
| CACNA1C | 12:2781443-2777666,-1 | kirare | exonic |
| CACNA1C | 12:2332647-2329703,-1 | swawnar | intronic |
| CACNA1C | 12:2716606-2712511,-1 | sweynar | intronic;promoter |
| CEP290 | 12:88448976-88452839,1 | smawnee | exonic;promoter |
| PAH | 12:103246691-103247638,1 | zeyglar | exonic;promoter |
| FOXG1 | 14:29234525-29194448,-1 | sachawbu | promoter |
| UBE3A | 15:25588526-25589187,1 | fluter | intronic |
| UBE3A | 15:25497370-25667967,1 | HBII-52-45 | exonic |
| UBE3A | 15:25614742-25620629,1 | palaby | intronic |
| CREBBP | 16:3915644-3917758,1 | hisuru | intronic |
| CREBBP | 16:3825300-3825806,1 | sturu | intronic;promoter |
| PAFAH1B1 | 17:2588915-2586599,-1 | gorwaw | exonic |
| PAFAH1B1 | 17:2509429-2508905,-1 | reyskey | intronic |
| NF1 | 17:29422468-29417805,-1 | jysa | exonic;promoter |
| NF1 | 17:29574230-29573746,-1 | skowybu | intronic |
| ADSL | 22:40784294-40768664,-1 | vawtey | intronic |
| SHANK3 | 22:51165983-51165307,-1 | chawvee | intronic |
| MID1 | X:10722039-10983726,1 | hero | intronic |
| NHS | X:17485009-17479794,-1 | flovar | intronic |
| NHS | X:17755214-17658171,-1 | kiro | exonic |
| NHS | X:17577248-17570467,-1 | shytor | intronic |
| DMD | X:33059409-33060012,1 | TBCAP1 | intronic |
| PQBP1 | X:48758712-48758117,-1 | foyker | exonic;promoter |
| ZNF81 | X:47701232-47700500,-1 | RPL7P57 | intronic |
| ZNF81 | X:47765952-47764918,-1 | zoyfoy | intronic |
| KDM5C | X:53198279-53200649,1 | morva | intronic |
| OPHN1 | X:67311255-67311837,1 | LOC100288917 | intronic |
| LAMP2 | X:119572593-119576511,1 | werkoy | exonic |
| FMR1 | X:147003676-146990949,-1 | ASFMR1 | intronic;promoter |
| MECP2 | X:153332348-153333135,1 | blufo | intronic |
| MECP2 | X:153307278-153307642,1 | tervar | intronic |
| MECP2 | X:153352646-153353089,1 | vyvar | intronic |
